# Role of surgery to the primary tumor in metastatic anaplastic thyroid carcinoma: pooled analysis and SEER-based study

**DOI:** 10.1007/s00432-022-04223-7

**Published:** 2022-08-12

**Authors:** Dmytro Oliinyk, Teresa Augustin, Josefine Rauch, Viktoria Florentine Koehler, Claus Belka, Christine Spitzweg, Lukas Käsmann

**Affiliations:** 1 Department of Radiation Oncology, University Hospital, LMU Munich, 81377 Munich, Germany; 2 Department of Internal Medicine IV, University Hospital, LMU Munich, 81377 Munich, Germany; 3 German Cancer Consortium (DKTK), Partner Site Munich, 80336 Munich, Germany

**Keywords:** ATC, Anaplastic thyroid carcinoma, Thyroidectomy, Surgery, Survival

## Abstract

**Purpose:**

Anaplastic thyroid carcinoma (ATC) is an orphan disease with a fatal outcome. Surgery to the primary tumor in metastatic ATC is controversial. Determination of specific surgical techniques may help facilitate local control and, hence, beneficial overall and disease-specific survival.

**Methods:**

Using individualized patient data derived from our systematic review of literature and our single center study (*n* = 123), conducting a Surveillance, Epidemiology, and End Results register (SEER)-based study (*n* = 617) we evaluated surgery, its combination with systemic and local therapies in metastatic ATC.

**Results:**

Pooled cohort study showed surgery (*p* < 0.001), RT ≥ 30 Gy (*p* < 0.001), ChT (*p* < 0.001) and multimodal treatment (*p* = 0.014) to result in improved OS univariately. In the multivariate analysis, surgery (1.997 [1.162–3.433], *p* = 0.012) and RT ≥ 30 Gy (1.877 [1.232–2.843], *p* = 0.012) were independent predictors for OS. In SEER-based study of patients undergoing any tumor-directed treatment (*n* = 445) total thyroidectomy (*p* = 0.031), administration of ChT (*p* = 0.007), RT (*p* < 0.001), combination of surgery and RT ± ChT (*p* < 0.001) and multimodal treatment (*p* < 0.001) correlated with an improved DSS univariately. On the multivariate analysis, debulking surgery was an independent predictor for a worse outcome (HR 0.535, 95%CI 0.332–0.862, *p* = 0.010), whereas RT administration correlated with a longer DSS (HR 2.316, 95%CI 1.362–3.939, p = 0.002). Among operated patients from SEER register total thyroidectomy (*p* = 0.031), ChT (*p* = 0.007), RT (*p* < 0.001), combination of surgery and RT ± ChT (*p* < 0.001) and multimodal treatment (*p* < 0.001) correlated with an improved DSS in the univariate analysis, whereas debulking surgery was inversely correlated with the DSS (*p* < 0.001). On the multivariate analysis, debulking surgery was an independent predictor for a worse DSS (HR 0.535, 95%CI 0.332–0.862, *p* = 0.010), whilst RT administration correlated with a longer DSS (HR 2.316, 95%CI 1.362–3.939, *p* = 0.002).

**Conclusions:**

Surgery to the primary tumor with the aim of R0/R1 resection, but not debulking, is associated with a significant OS and DSS benefit even in systemically metastasized disease.

**Supplementary Information:**

The online version contains supplementary material available at 10.1007/s00432-022-04223-7.

## Introduction

Anaplastic thyroid carcinoma (ATC) is an orphan disease and one of the most aggressive cancers due to its rapid progression with limited mean survival of 3–6 months (Neff et al. 2008). Distant metastases that are often present at the time of initial diagnosis in ATC do not only result in a very dismal prognosis but also present a major challenge in decision making for the optimal treatment regime (Maso et al. 2017; Maniakas et al. 2020). At this stage most commonly utilized therapies include cytotoxic chemotherapy (ChT) with or without radiation therapy (RT) (Bible et al. 2021; Haddad et al. 2018; Filetti et al. 2019). More recently immunotherapeutic approaches and targeted therapies have been proposed for the treatment of advanced and metastatic ATC if targetable mutations are detected (Bible et al. 2021; Haddad et al. 2018; Filetti et al. 2019; Subbiah et al. 2018).

Current guidelines (National Comprehensive Cancer Network [NCCN], American Thyroid Association [ATA]) recommend to consider thyroidectomy also in ATC stage IVC if the primary tumor is considered resectable and R0/R1 margins are achievable (Bible et al. 2021; Haddad et al. 2018). It is important to consider local and distant complications when planning therapy at this stage of the disease and to evaluate individual susceptibility toward a chosen therapy within a multidisciplinary expert team. Surgical therapy to the primary tumor ranging from debulking surgery to R0 thyroidectomy could possibly prevent patients from developing life-threatening aspirations, dysphagia, dyspnea, bleedings or superior vena cava syndrome (Haigh et al. 2001). Some of these complications may, however, be prevented by less invasive interventions, e.g., tracheostomy, percutaneous endoscopic gastrostomy (PEG) or venous stenting. Furthermore, sufficient radiation therapy to the primary site or systemic therapies with platin-based agents or targeted therapies to actionable mutations such as *BRAF V600E*, or *NTRK* and *RET* gene fusions may also provide sufficient local control rates (Filetti et al. 2019). Thus, it is important to analyze outcomes and prognostic factors among patients with metastatic ATC to determine the rationale for an invasive intervention.

In this study we aim to investigate the role of different surgical procedures to the primary tumor in patients with ATC stage IVC on their survival. Hereby we compared various surgery types among each other by performing a systematic review of literature, pooled analysis derived from individualized patient data and SEER-database analysis.

## Patients and methods

### Systematic review of literature and pooled analysis

**Fig. 1:**
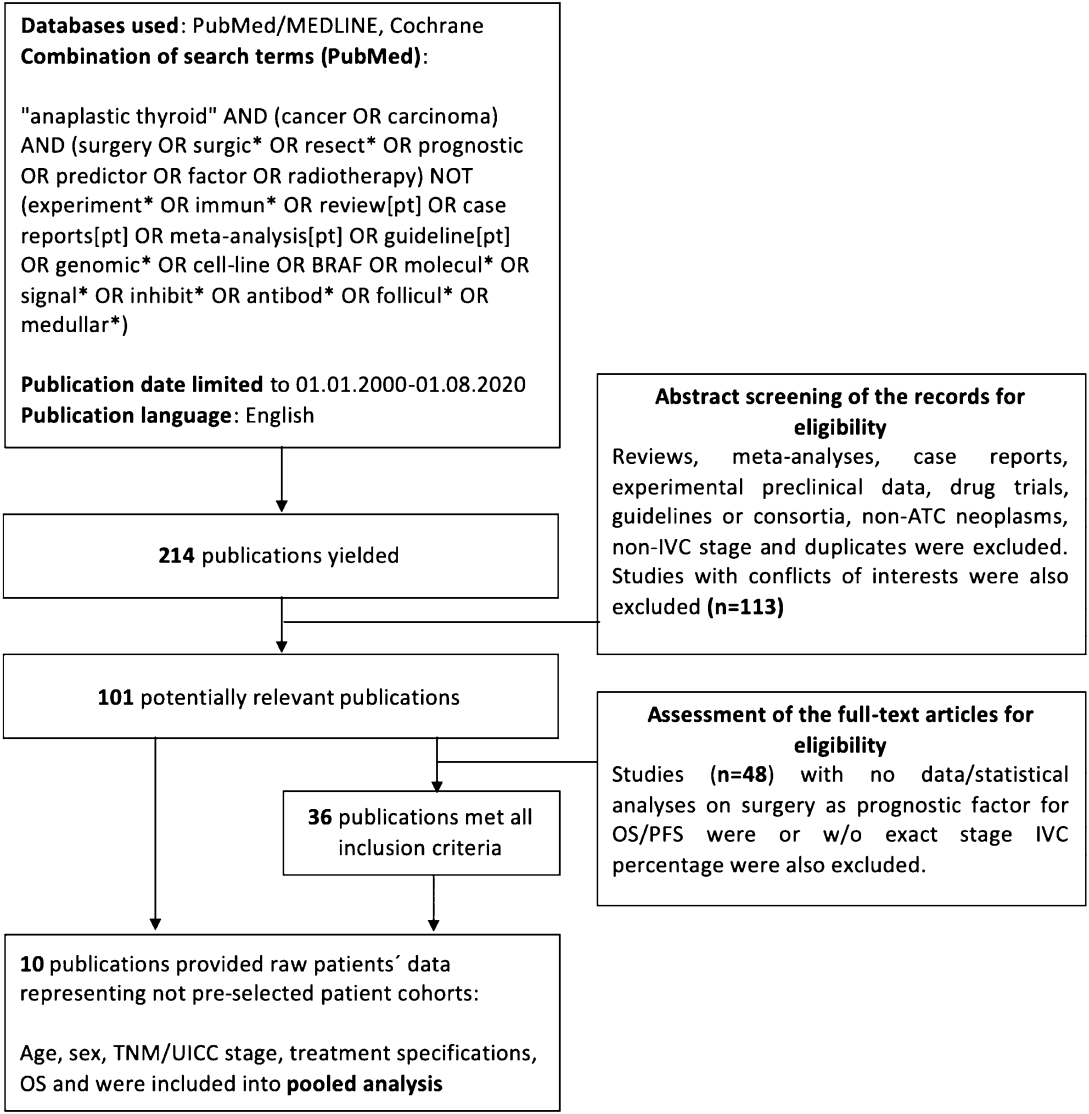
PRISMA-flowchart for the systematic review of literature: surgery in stage IVC ATC patients

A systematic review of literature was primarily carried out on 30 th October 2020 and repeated on 15 August 2021 using PubMed/MEDLINE (National Center for Biotechnology Information, Bethesda, MD, USA) and Cochrane databases to identify relevant publications according to PRISMA-protocol. A full list of search terms is provided in Fig. 1. Articles published in English, in the timeframe from 1 January 2000 to 1 August 2021 and identified by the mentioned terms were included into the preliminary analysis. Further eligibility assessment incorporated abstract screening for article and neoplasm type, as well as duplicates. Studies with conflicts of interests, e.g. publications of our facility were excluded. Reviews, meta-analyses, case reports, experimental preclinical data, drug trials, guidelines or consortia and studies without stage IVC ATC were excluded. Remaining articles were analyzed in full-text and excluded, if data or statistical analyses on surgery as prognostic factor for OS/PFS were missing. Furthermore, only studies with exact stage IVC percentage data were included. Publications with individualized patients’ data on age, sex, TNM/UICC stage, treatment specifications and OS were included into pooled analysis. The complete review process was based on the PRISMA guidelines and is depicted in Fig. 1.

A cohort of patients with metastatic ATC from our facility has been described previously and pooled with the newly identified data to form a representative cohort (Augustin et al. 2021). Inclusion and censorship criteria have been reported in that study.

### SEER-based analysis

Based on the promising results of the pooled analysis we used Surveillance, Epidemiology, and End Results (SEER) database to further verify our null hypothesis. Data on all patients diagnosed with ATC employing histopathological codes of International Classification of Disease for Oncology (3rd edition; ICD-O-3; code 8021/3 [Carcinoma, anaplastic, NOS] and site [Thyroid]) from 2000 to 2016 were analyzed. Only patients with metastatic ATC were included into our study. Patients with aberrant stages as M1/IVC were excluded from the analysis. Staging was based on the SEER histological stage A (1973–2015) [Distant] and American Joint Committee on Cancer (AJCC) 3^rd^–7^th^ editions [M1/IVC]. Following information was subsequently extracted from the SEER database: sex, age, surgery record and type according to “Rx Surg Prim Site (1998+)”, RT codes and sequences, ChT records, cause-specific deaths codes, vital status and survival in months. Data acquisition date was 2 August 2021. Surgery records “isthmectomy only”, “resection of less than one lobe” and “local surgical excision” were considered as debulking surgery. Patients without specified type of thyroidectomy “thyroidectomy NOS”, “surgery NOS” or “unknown if surgery performed; death certificate ONLY” were excluded from the analysis.

### Statistical analysis

Statistical analysis was conducted using SPSS statistics 25 (IBM, Chicago, IL, USA). Univariate analysis of variables comprised log-rank test. Significant variables were subsequently analyzed multivariately in the Cox regression. Significance level was defined for all analyses at α = 0.05. In our single-center data patients were censored if lost to follow-up, overall survival (OS) was defined as the time from initial diagnosis to death, sufficient RT dose was defined as a total RT dose of  ≥ 30 Gy due to a considerable number of patients receiving a total dose of < 30 Gy. In these cases, RT was mostly discontinued given the premature patients death or local complications.

## Results

### Systematic review of literature and pooled analysis

In total, 214 publications were yielded by our combination of search terms in the PubMed MEDLINE database. Cochrane search did not reveal any relevant studies. Potentially relevant publications accounted for 101 studies and were identified by abstract screening for eligibility, as shown in Fig. 1. Full-text assessment of these articles was based on the presence of statistical analyses specific for our hypothesis, namely, surgery as prognostic factor for improved overall or progression-free survival (OS, DSS and PFS, respectively). Thus, 36 retrospective studies were fully included into our systematic review (Table 1). A total of 12,725 patients were evaluated by the mentioned studies. Stage IVC accounted for 41.1% or approximately 5226 of these patients. Resection margins were reported at least partially by 16 (44.4%) of the studies. Twenty-seven (75%) publications showed surgery or certain resection status to be favorable for a longer survival, decreased relative risk or an improved local control (LC) in the univariate analysis. Two studies claimed their univariate analysis to be significant at α-levels of *p* < 0.2 or did not provide additional information on the significance level other than *p* < 0.1. In multivariate analysis, surgery or certain resection status were identified as an independent prognostic factor in 19 (52.7%) publications. Two publications did not provide *p* values for their multivariate analyses, but the 95% CI did not cross HR value of 1.0.

**Table 1:** Systematic review of literature—surgery in ATC

Author	Number of patients	Median age (IQR, years)	Stage IVA/IVB/IVC %	Surgery	R status	RT % and regimen	Total RT dose	ChT %	ChT agent	Additional information	Univariate for surgery (HR [95% CI]; *p* value)	Multivariate for surgery (HR [95% CI]; *p* value)
Fan et al. (2020)	104	63.5 (28–87)	4.8/73.1/22.1	NOS—50%	R0—12.5% R1—21.2% R2—16.3%	100.0	Median 66 Gy/33fr (6–70.25 Gy/3–40)	95.2—concurrent	DOX-based—73.7% PTX-based—24.3% Others—2%	Trimodal Therapy—51.0%	*p* < .001	0.278 [0.029–2.687]; *p* = 0.269. NS
Corrigan et al. (2019)	28	70.9 (63.8–74.7)	7.1/74.1/17.9	NOS 71.4%	NR	67.9	< 40 Gy—39.3% ≥ 40 Gy—21.4	50%	*With RT:* DOX—36.4% Other combinations—63.6%	S + RT + ChT—35.7% S + RT—17.9% RT + ChT—10.7% RT only—10.7% S only—17.9% ChT only—3.6%	0.384 (0.157–0.938), *p* = .036	0.198 (0.065–0.598), *p* = *.004*
Glaser et al. (2016)	3552	≥ 65—68.4%	NR/NR/41.6	TT—23.2% Other—26.3%	Margins negative—13.2% Margins positive/unknown—36.3%	58.7	Median 45 Gy (≤ 36 Gy—32.1%; ≥ 59.4 Gy—38.1%)	46.1	Single agent—57% Multiagent—43%	NR	S other than total: 1.32 (1.13–1.54), *p* < 0.005	OR for death within 2 y: 0.59 (95%CI 0.45–0.78), *p* < 0.005
Paunovic et al. (2015)	150	< 40—1.3 41–50—6.1 51–60—19.3 61–70—54.0 > 70—19.3	NR/NR/32.9	Tumor reduction—31.8 Lobectomy—4.7 Thyroidectomy and dissection—47.1	NR	81.2	NR	2.4	NR	NeoAd RT—2.4 Adj RT—78.8	RR 1.51 (1.30–1.81), *p* < .001	RR 0.43 (0.29–0.63) p < .001
Takahashi et al. (2019)	33	68 (41–87)	12/39/39	NOS—39%	NR	100	Median 50 Gy (5–74 Gy)	52	CARB + PTX—35.3% NDP + 5-FU—17.6% NDP + ETOP—11.8% PTX—11.8% Other—23.5% N = 17	CRT—45% N = 33	NS OS: 0.77 (0.35–1.66), *p* = .510 LC: 0.63 (0.18–2.07), *p* = .445	NR
Wendler et al. (2016)	100	70.5 (38–92)	9/32/54	Primary—83%: - TT—43% - HT—19% - Two-stage thyroidectomy—8% Tumor reduction—3%	R0—14% R1—27% R2—52%	83	Median 57.6 (13.5–80) Gy	56	DOX—25 PTX—9 PTX + PEM—8 DOX + CDDP—8 CARB + PTX—14 TKI´s—10 Other—10 (*absolute numbers as in the study*)	S + ChT + EBRT—49% S + EBRT—19% S only—11% EBRT only—9% EBRT + ChT—4% S + ChT—4% ChT only—0% Best supportive care—4%	NS, *p* = .376	NR
Jacobsen et al. (2017)	31	69 (26–87)	6.5/64.5/29	Any S—100% Thyroidectomy—69.2%	R0—38.5% R1—46.15% R2—15.4%	100	Median 64 (41.6–64) Gy	74.2	DOX-based—74.2%	RT Only—19.3% CRT—38.7% S + RT—6.5% S + CRT—35.5%	No S: 4.95 (2.01–12.2), *p* < .2	No S: 4.78 (1.92–11.9), *p* < .2
Bhatia et al. (2009)	53	Median NR; Mean: 66.1 ± 12.0	NR/NR/47.2	Complete—35.8% Subtotal—22.6%	NR	100	3DRT: 46.5 (4.0–70.0) Gy IMRT: 60.0 (39.9–69.0) Gy	91 (81% concurrent)	CARB/PTX—18.8% DOX/CDDP—16.7% CDDP—14.6% CARB—10.4% CDDP/ FLD/ARA-C—4.2% DOX/CDDP/PTX—4.2% Other combinations—12.5%	NR	NS 0.57 (0.31–1.05), *p* = .07	NR
Kim et al. (2007)	121	Median NR; Mean: 64 ± 11	NR/NR/24	Bilateral—59% Unilateral—12%	NR	RT only—45.5 CRT—13.2	NR	20.7%	DOX + CDDP—88% GEM—8% DOX—4%	RT only—10.7% ChT only—1.6% ChT + RT—4.1% S only—20.7% S + RT—34.7% S + ChT—5.9% S + ChT + RT—9.1% BSC—13.2%	*p* < .001	NS
Brignardello et al. (2014)	55	73.15 (61.61–79.19)	0/43.6/56.4	74.5%: maximal debulking—70.1% Partial debulking—29.3%	NR	54.5	50–54 Gy in 1.8–2.0 Gy/fr	87.3	DOX and CDDP-based—87.5% PTX-based—12.5%	Early S alone—12.2% S + ChT—29.3% S + ChT + RT—58.5% NeoChT + RT + S—9.8% NeoChT + RT—4.9% NeoChT only—14.6%	Partial S: 5.33 (2.33–12.19), *p* < .001	Partial S: 5.36 (2.34–12.30), *p* < .001
Pezzi et al. (2017)	1288	Median NR; Mean—70.2	NR/NR/47	Any S—11.7%	NR	47.7	< 45 Gy—47.9% 45–59.9 Gy—21.8% 60–75 Gy—29.0% NR—1.3%	36.6	NR	NR	*p* = .004	0.786 (0.643–0.962), *p* < .001
Liu et al. (2016)	50	≤ 60—52% > 60—48%	30/38/32	100%	R0—34% R1—42% Other—24%	32	30–70 Gy—100% > 40 Gy—93.8%	16	IFX, DOX, DTIC	S only—42% S + RT/ChT—34% CRT—2% ChT only—4% Tracheostomy, biopsy, other—18%	R0&R1 vs. No S in ATC IVA/B: *p* < .001 S vs. non-S-treatment in ATC IVC: *p* = .521	S in ATC IVA/B: Exp(B) = .331, *p* = .038
Pierie et al. (2002)	67	73 (40–92)	NR/NR/49	Any S—65.7%: Complete—27.3% Incomplete—72.7%	NR	83.5	25–73 Gy: ≤ 45 Gy—51.8% > 45 Gy—48.2%	31.3	DOX-based—NR	NR	No S: 28.9 (CI—NR), *p* < .0001	Any S: 0.28 (CI—NR), *p* = .0004
Aslan et al. (2014)	29	64.5 (35–91)	10.3/62.1/27.6	Any S—55.2%: Total thyroidectomy—56.3% Subtotal—25% Biopsy—18.7%	R0—25% R1—25% R2—50%	65.5	NR	44.8	DOX—84.6% DOX + CDDP—7.7% (1) DOX + SOR—7.7% (1)	S + RT + ChT—31% S + RT—17.2% RT + ChT—13.8% RT only—3.5% (1) S only—10.3% None—24.1%	R0: *p* = .05	NR
Kihara et al. (2004)	19	Median NR; Mean: 73.4 (45–87)	NR/NR/47	Any S—53%: Complete—40%	R0—40% R1/2—NR	68.4	≥ 45 Gy—69.2% < 45 Gy—30.8%	63.2	DOX (alone/combination)—75% PTX—8.3% (1) Other—NR	S + RT + ChT—26.3% S + RT/ChT—10.5% S alone—15.8% Other—NR	Complete/no S: *p* = .002	Incomplete resection: RR 1.475 (1.168–16.340), *p* = .0284
Zivaljevic et al. (2014)	150	Median NR; (35–89) ≤ 60—26.7% > 60—73.3%	NR/NR/32.9	Any S—56.7%: Lobectomy—4.7% Thyroidectomy and dissection—47.1% Tumor reduction—31.8% Biopsy—14.1% Tracheotomy—2.4%	NR	Pre/Post S: 46	NR	1.3%	NR	NR	*p* < .1	OR = 0.43 (0.29–0.63), p < .001
Huang et al. (2019)	735	70 (60–80)	NR/NR/44.1	Any S—44.2%: TT—60% Less than TT—40%	NR	54.7	NR	42.4	NR	No treatment—21.9% ChT only—3.7% S only—16.3% S + ChT—2.6% RT only—11% RT + ChT—17.2% S + RT—7.7% S + RT + ChT—19.5% Total: 725 patients (Table 2)	*p* < .001	TT vs. Less than TT: 0.655 (0.521–0.838), *p* = .001)
Yau et al. (2008)	50	72 (36–104)	NR/NR/18	Any S—68%: TT—82.4% Subtotal—8.8% Lobectomy—5.9% Radiofrequency ablation—2.9% (1)	NR	46	NR	36	NR	NR	*p* < .01	NS
Sun et al. (2015)	42	60 (42–80)	7.1/69.1/23.8	Any S—78.6%: Thyroidectomy alone—60.1% Thyroidectomy + neck dissection—38.9%	NR	100	24–70 Gy < 40 Gy—47.6% ≥ 40 Gy—52.4%	28.6	DOX + CDDP—25% PTX + CDDP—33.3% BLEO, 5-FU + CDDP—41.7%	ChT alone—4.8% S alone—11.9% RT alone—28.6% S + RT—26.2% S + RT + ChT—14.3% S + ChT—9.5% No treatment—4.8%	3-year OS for S type: *p* = .113	NR
Lee et al. (2016b)	98	Median NR; 63.5–13.4	NR/NR/20.4	NOS—58.2%	NR	17.3	NR	7.1	NR	NR	Resectability: OR: 2.57 (1.60–4.15), *p* < .001	Resectability: Adjusted OR: 1.39 (1.21–1.74), *p* = .004
Haigh et al. (2001)	33	69 (47–80)	NR/NR/64	Any S—79%: TT—54% Near-total thyroidectomy—19% Total thyroid lobectomy—12% Minor excision/isthmusectomy—8% Node dissection alone—8% Concomitant neck dissection—31% Concomitant tracheostomy—8%	NR	NR	45–75 Gy	NR	DOX, PTX, CDDP, CARB, ETOP, CYC, MEL, BLEO	NR	Curative S vs. Palliative S: *p* < .001	Curative S: RR 0.1 (0.02–0.63), *p* = .01
Baek et al. (2017)	329	Median NR; 68.1 (22–89)	17.3/49.5/31.3	Any S—57.1%: Curative S—50% Curative S + CRT/RT—44.7% Curative S + ChT—5.3%	R0—11.7% R1—71.3% NR—17%	40.7	< 40 Gy—24.6% ≥ 40 Gy—75.4%	NR	CDDP, CARB, DTX, PTX, DOX	No definite treatment—24.6% RT/concurrent CRT—15.2% Curative resection—28.6% Curative resection and adjuvant RT/concurrent CRT—25.5% Curative resection and adjuvant chemotherapy—3% Chemotherapy—3%	Treatment method including S: *p* < .01	Curative S only: 0.505 (0.339–0.752), *p* < .01
Dumke et al. (2014)	40	67 (38–84)	NR/NR/22.5 (at diagnosis)	Any S—80%: TT—68% Subtotal—12%	R0—10% R1—7.5% R2—50% Rx—32.5%	97.5	Median 50 (6–60.4) Gy	15	Mono DOX—2patients PTX mono/ combination IFX/CARB/ ETOP, EPR mono + unknown protocol—á 1 patient	NR	R-status: *p* = .41	NR
Mohebati et al. (2014)	83	60 (28–89)	NR/NR/24	NOS—83%	R0/1—40.6% R2/x—59.4%	assured 64	6–70 Gy	assured 57	DOX ± Pt-based ChT	S ± RT ± ChT—71% S alone—12% ChT/RT—5% RT alone—4% S + RT—14% S + PORT + ChT—46% S + ChT—11%	*p* < .001	2996 (1.2–7.1), *p* = .013
Kebebew et al. (2005)	516	Median NR; Mean 71.3 ± 12.7	NR/NR/43	Any S—49%: Total—4.7% Subtotal or near TT—14.6% Lobectomy and/or isthmusectomy—9.1% Removal < lobe—3.2% Thyroidectomy, NOS—2 p Surgery, NOS—67.6%	NR	63.2	NR	NR	NR	NR	*p* < .0001	NS 0.779 (0.312–1.946)
Akaishi et al. (2011)	100	68 (41–90)	11/31/58	Any S—70%: TT—50% Subtotal—15.7% Lobectomy—32.9% Partial lobectomy—1.4% (1)	R0—34.3% R1/2—65.7%	78	≥ 40 Gy—74.4% < 40 Gy—25.6%	28	PTX, CDDP, DOX, ETOP-based	S + CRT—15% S + RT—60% CRT—5% RT alone—10% ChT alone—10%	*p* < .0001	No S: RR 3.99 (2.37–6.66), *p* < .0001
Derbel et al. (2011)	44	65 (44–80)	NR/NR/45.4	Any S—100%: TT—63.6% Near total thyroidectomy—15.9% Biopsy/Debulking—20.4%	NR	88.5	46–50 Gy	86.4	DOX + CDDP—86.8% DOX + CARB—7.9% DOX—2.6% (1) PTX—2.6% (1)	S alone—4.5% S + ChT—7% S + RT + ChT—79.5% RT alone—4.5% S + RT—4.5%	Near-total/total S: *p* < .05	NR
Crevoisier et al. (2004)	30	Median NR; Mean 59 (40–79)	NR/NR/20	Any S—80%	MCR—58.3% IR—41.7%	100	40–55 Gy	100	DOX (60 mg/m2) + CDDP (120 mg/m2)—100%	NR	*p* = .01	MCR: 2.7 (1.1–6.8), *p* = .04
Haymart et al. (2013)	2742	Mean: 70 ± 12.29	37.6/37.3/25.1 (n = 649)	Any S—51.4% (*n* = 2086): Total thyroidectomy—64% Lobectomy—26%	NR	60.4 (n = 2086)	NR	40% (n = 2086)	NR	NR	*p* < .001	No S: UHR 1.85 (1.67, 2.05) AHR 1.79 (1.61, 1.99)
McIver et al. (2001)	134	Mean: 60	NR/NR/46	Any S—72%: TT—13% Near-total—29% Lobectomy—46% Complex—12%	Complete—30% Minimal residue—26% Gross incomplete—44%	50	NR	12	NR	S + RT—39.6% S + ChT—9.4%	NS *p* > .4	NR
Sugitani et al. (2012)	677 (547 with common type ATC)	Mean: 68.7 ± 11.0	13/44/42	Any S—55%: Palliative—51.8% Radical—48.2%	NR	58.3	< 40 Gy—75.2% ≥ 40 Gy—24.6%	46.6	NR	NR	*p* = .0002	Radical S: 0.43 (0.27–0.68), *p* = 0003
Brignardello et al. (2007)	27	46–92	NR/NR/55.6	Any S—74.1%:Maximal debulking—70% Palliative—30%	NR	55.6	36–40 Gy	55.6	DOX, CDDP, PTX	NR	Maximal debulking: 0.18 (0.06–0.54)	Maximal debulking: 0.23 (0.07–0.79)
Ridder et al. (2020)	812	73 (29–99)	NR/NR/42.1	NOS—31%	NR	52	NR	11	NR	Only S/RT/ChT—40% S + RT/CRT/S + ChT/ RT + ChT—20% S + CRT/S + RT + ChT—4%	0.4 (0.3–0.5), *p* < .001	0.5 (0.4–0.6), *p* < .001
Roche et al. (2010)	26	Mean 72.1 (52.3–90.8)	NR/NR/15.4	Any S—84.6%: TT/subtotal—72.7% Lobo-isthmectomies/hemi-thyroidectomies—18.2% Tumorectomy—9.1%	R0—46.2% R1—53.9% (*n* = 26)	53.8	NR	19.2	NR	Adjuvant RT—50% S + RT + ChT—19.2%	R0/R1 *p* = .014	NS
Gui et al. (2020)	1014	≥ 65—64.5% < 65—35.5%	NR/NR/56.1	Any S—45.5%: TT—61.9% Lobectomy—38.1%	NR	56.3	NR	NR	NR	NR	Total: 0.705 (0.570–0.873), *p* = .001	Total: 0.782 (0.630–0.971), *p* = .026
Ito et al. (2012)	40	74 (52–85)	0/62.5/37.5	Any S—50% TT—40% Subtotal/near-total—20% Palliative/tracheostomy—40%	R0—0% R1—10% R2—90%	70	45–60 Gy	52.5	Combinations of CDDP, ADM, EPR, ETOP, DTX, 5-FU and PTX	NR	S + CRT beneficial for median survival time: *p* < .01	NR

*S* surgery, *ChT* chemotherapy, *RT* radiation therapy, *CRT* concurrent chemoradiotherapy, *TT* total thyroidectomy, *HT* hemithyroidectomy, *NOS* not otherwise specified, *NR* not reported, *R* resection status, *IQR* interquartile range, *HR* hazard ratio, *RR* relative risk, *OR* Odds ratio, *CI* confidence interval, *OS* overall survival, *LC* local control, *EBRT* external beam radiation therapy, *NeoAd* neoadjuvant, *Adj* adjuvant, *fr*. fraction, *Gy* Gray, *DOX* doxorubicine, *PTX* paclitaxel, *CARB* carboplatin, *NDP* nedaplatin, *5-FU* 5-fluoruracil, *ETOP* etoposide, *PEM* pemetrexed, *CDDP* cisplatin, *TKI* tyrosine kinase inhibitor, *FLD* fludarabine, *ARA-C* cytarabine, *CYC* cyclophosphamide, *MEL* melphalan, *BLEO* bleomycin, *SOR* sorafenib, *DTIC* dacarbazine, *IFX* ifosfamide, *GEM* gemcitabine

**Fig. 2:**
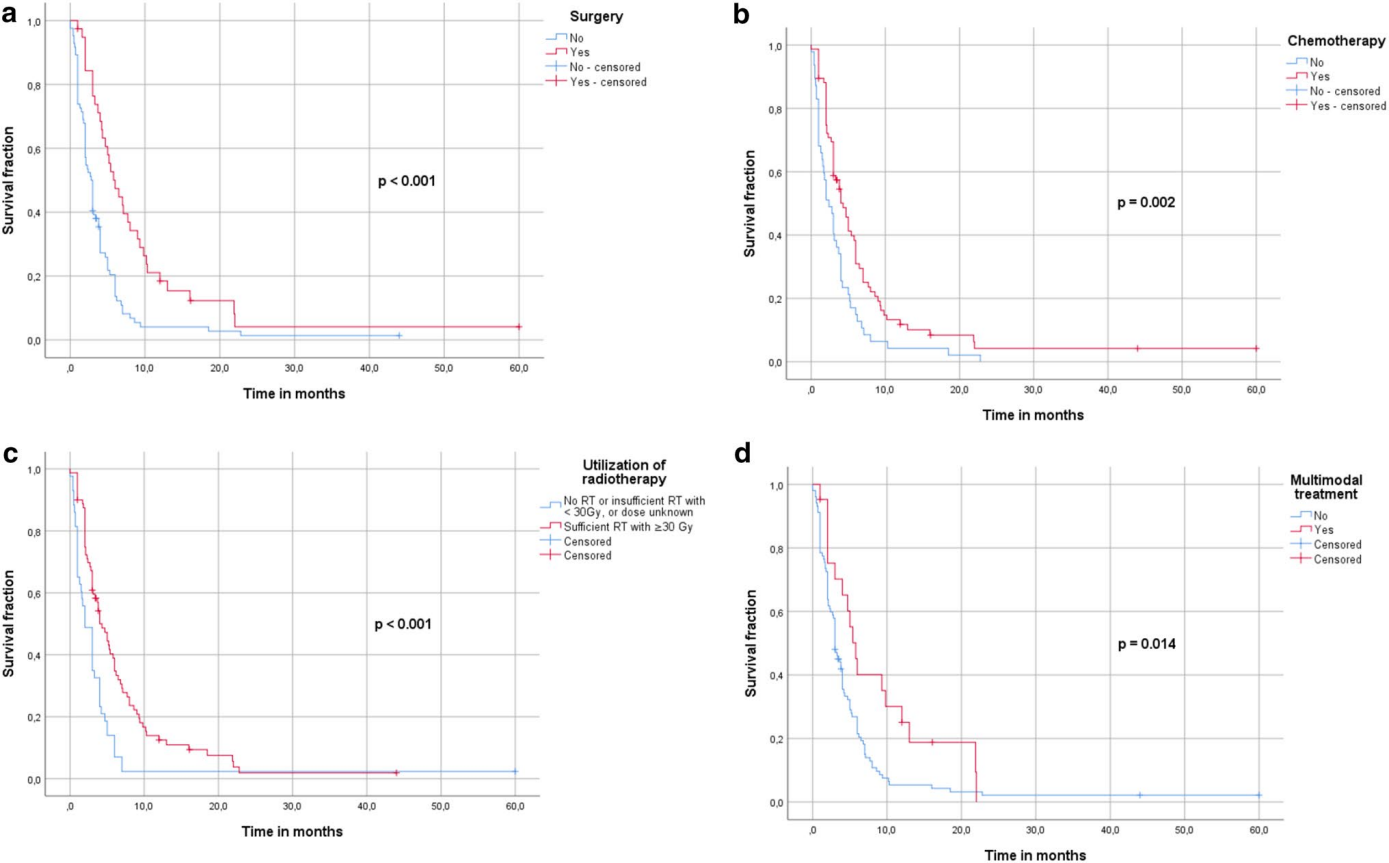
**A**–**D**. Univariate analysis of prognostic factors for patients from the pooled cohort

In total, 10 studies (Takahashi et al. 2019; Aslan et al. 2014; Crevoisier et al. 2004; Stavas et al. 2014; Ito et al. 2012; Lim et al. 2012; So et al. 2017; Tennvall et al. 2002; Troch et al. 2010; Busnardo et al. 2000) provided individualized patient data that were eligible for a pooled analysis with our single center cohort described previously (Augustin et al. 2021). This new cohort included 123 patients with stage IVC ATC and a median age of 71 (39–94) years (Table 2). Treatment records were available for RT, ChT and surgery in 61.8, 76.4 and 31.7% of the cases, respectively. Treatment sequences with RT/ChT were reported for 96.7% of the population. In 39.8% of the cases, concurrent chemoradiotherapy (CRT) was administered. Sufficient RT was used in 33 (26.8%) cases. Surgical intervention included debulking, sub- or near total thyroidectomy, total thyroidectomy or was not specified. Information about resection status was available for 35 (28.4%) of these patients and multimodal treatment was applied in 21 (17.1%) of the cases. OS rate was 27.1% and 7.9% at 6 and 12 months, respectively. In the univariate analysis surgery (*p* < 0.001), administration of sufficient RT ≥ 30 Gy (*p* < 0.001), ChT (*p* < 0.001) and multimodal treatment (*p* = 0.014) resulted in improved OS (Fig. 2A–D). A specific age group or the age < 65 years did not correlate with an improved OS, but there was a tendency for patients < 65 years towards a better survival rate at 6 months: 19.3 vs. 27.1%. In the multivariate analysis, only surgery (1.997 [1.162–3.433], *p* = 0.012) and sufficient RT ≥ 30 Gy (1.877 [1.232–2.843], *p* = 0.012) were identified as independent predictors for OS (Table 3).

**Table 2:** Patients characteristics from the pooled analysis: whole cohort and only operated patients

Characteristic	Number and %	Number and %
Age in years (median, IQR)	71 (39–97)	68 (39–83)
	*n* = 123	*n* = 39
Age group		
< 50 years	5 (4.1%)	3 (7.7%)
50–59 years	19 (15.4%)	6 (15.4%)
60–69 years	28 (22.8%)	11 (28.2%)
70–79 years	50 (40.7%)	18 (46.2%)
≥ 80 years	21 (17.1%)	1 (2.6%)
Elderly patients		
≥ 65 years	40 (67.5%)	24 (61.5%)
< 65 years	83 (32.5%)	15 (38.5%)
Sex		
Female	71 (57.7%)	22 (56.4%)
Male	44 (35.8%)	15 (38.5%)
Not reported	8 (6.5%)	2 (5.1%)
	*n* = 123	*n* = 39
Stage		
IVC	100%	100%
ChT record	76 (61.8%)	33 (84.6%)
RT/ChT sequence		
ChT concurrent to RT	49 (39.8%)	23 (39.8%)
ChT not concurrent to RT	11 (8.9%)	6 (8.9%)
ChT without RT ± surgery	12 (9.8%)	2 (9.8%)
RT without ChT ± surgery	29 (23.6%)	4 (23.6%)
No ChT and no RT ± surgery	18 (14.6%)	2 (14.6%)
Sequence unknown	4 (3.3%)	2 (3.3%)
RT record	97 (76.4%)	35 (89.7%)
Sufficient RT ≥ 30 Gy	80 (*82.5%)	33 (*94.3%)
RT dose unknown	2 (*2.1%),	1 (*2.85%),
	*n = 97	*n = 35
Surgery	39 (31.7%)	39 (100%)
Surgery type	*n* = 39	–
Total thyroidectomy	11 (28.2%)	
Subtotal OR near-total thyroidectomy	4 (10.3%)	
Lobectomy/hemi-thyroidectomy	2 (5.1%)	
Debulking	5 (12.8%)	
N/A	17 (43.6%)	
Resection margins	*n* = 39	–
R0	2 (5.1%)	
R1	17 (43.6%)	
R2	16 (41.0%)	
N/A	4 (10.3)	
Margins < R2	*n* = 39	–
Yes	16 (41.0%)	
No	19 (48.7%)	
N/A	4 (10.3%)	
Multimodal treatment	21 (17.1%)	21 (53.8%)
Total number of patients	123	39

**Table 3:** Uni- and multivariate analyses of the pooled cohort

Cohort	Univariate analysis	Multivariate analysis (HR, 95%CI, *p* value)
**Total**	–	–
Surgery	***p* < 0.001**	**1.997 [1.162–3.433], *p* = 0.012**
ChT record	***p* = 0.002**	1.479 [0.982–2.228], *p* = 0.061
Sufficient RT ≥ 30 Gy vs. No RT/RT < 30 Gy/dose NA	***p* < 0.001**	**1.877 [1.232–2.843], *p* = 0.012**
Multimodal treatment with RT ≥ 30 Gy	***p* = 0.014**	0.772 [0.384–1.555], *p* = 0.469
Age cohorts	*p* = 0.844	–
Elderly (≥ 65 years)	*p* = 0.65	–

Statistically significant values (*p*< 0.05) are in bold

**Fig. 3:**
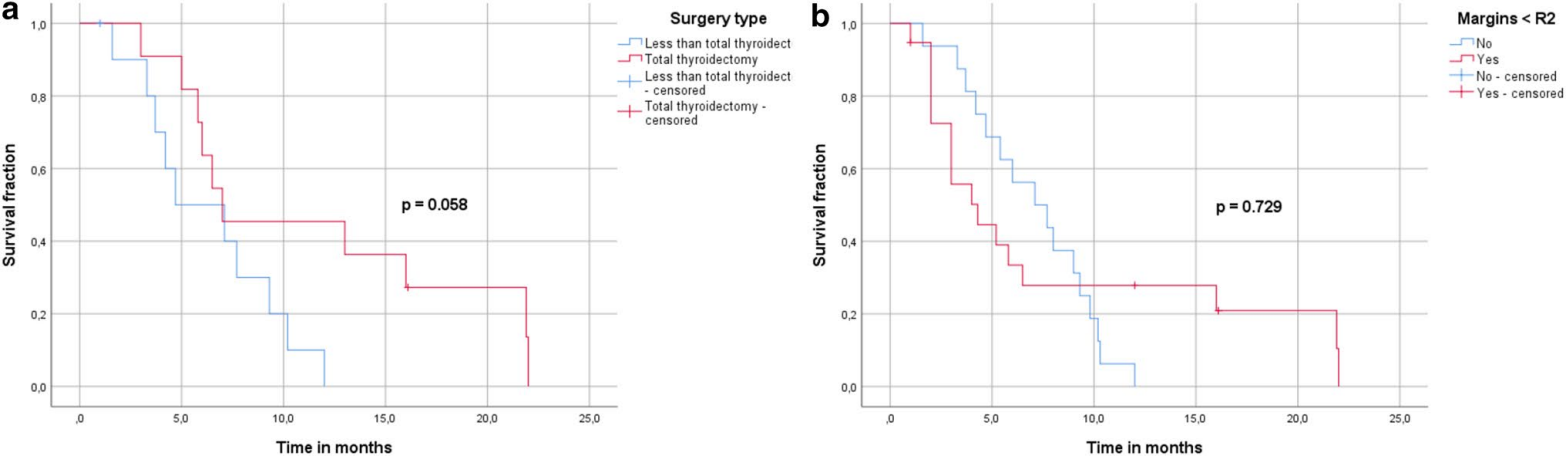
**A**–**B**. Univariate analysis of prognostic factors within the operated cohort from the pooled analysis

To identify the most favorable surgical approach, we selected or identified patients with ATC stage IVC who underwent different surgical procedures, and analyzed possible predictors for an improved OS. A sub-cohort of 39 patients was investigated (Table 2). Median age was 68 (39–83) years, 84.6% of the patients received RT ≥ 30 Gy, 39.8%—with concurrent ChT, and multimodal therapy was administered to 53.8%. Surgical types analyzed included total thyroidectomy in 28.2% of the cases, sub- or near total thyroidectomy, lobectomy/hemi-thyroidectomy and debulking in 10.3, 5.1 and 12.8% of the cases, respectively. Surgical data of 43.6% of the operated patients was, unfortunately, not available. Resection margins other than R2 were reported for 41% of the patients. In the univariate analysis only, total thyroidectomy showed a tendency towards an improved OS, when compared to other surgery types with survival rates at 9 months of 45 and 30%, respectively (*p* = 0.058) (Fig. 3A, B). Margin status, therapy regimen, age or multimodality were not significantly correlating with OS in the operated sub-cohort.

Given the insufficiency of the individual surgical data gathered from the pooled analysis we decided to use SEER data base to identify best surgical approach. Therefore, we have analyzed a cohort of 617 ATC stage IVC patients (Table 4). Median age was 69 (33–97) years, ChT and RT were employed in 61.4 and 52% of patients, respectively. A total of 187 (30.3%) patients underwent surgery, i.e., either lobectomy and/or isthmectomy, removal of less than one lobe, sub- or near-total thyroidectomy and total thyroidectomy in 22.4, 18.2, 11.8 and 47.6% of cases, respectively. Multimodal treatment was administered to a total of 11.7% of patients and > 75% of all patients had an ATC-related death. On the univariate analysis surgery (*p* < 0.001), administration of ChT (*p* < 0.001) or RT(*p* < 0.001), multimodal treatment (*p* < 0.001) and age group (*p* < 0.001) correlated with an improved OS and DSS. Age ≥ 65 years (OS: *p* < 0.001, DSS: *p* = 0.008) correlated with a worsened OS and DSS (Suppl. Figures 1A–F, 2A–F). On the multivariate analysis surgery (OS–HR 1.934, 95% CI 1.538–2.427, *p* < 0.001; DSS–HR 1.803, 95% CI 1.275–2.550, *p* < 0.001), RT (OS–HR 1.873, 95% CI 1.558–2.247, *p* < 0.001; DSS–HR 1.611, 95% CI 1.191–2.178, *p* = 0.002), ChT (HR 1.727, 95% CI 1.412–2.114, *p* < 0.001; DSS–HR 1.572, 95% CI 1.197–2.064, *p* = 0.001) were independent predictors for an improved OS and DSS (Table 5). Age ≥ 65 years (HR 0.795, 95% CI 0.665–0.950, *p* = 0.012) was also identified as an independent predictor for a higher overall mortality. Administration of best supportive care only correlated inversely with DSS on the univariate analysis (*p* < 0.001), but it was not an independent predictor for a worse DSS.

**Table 4:** Patient characteristics from SEER database (any treatment left, without best supportive care mid, operated right)

Characteristic	Number and %	Number and %	Number and %
Age in years (median, IQR)	69 (33–97)	67 (34–93)	66 (34–93)
Age group			
< 50 years	44 (7.1%)	40 (9.0%)	22 (11.8%)
50–59 years	95 (15.4%)	78 (17.5%)	34 (18.2%)
60–69 years	176 (28.5%)	134 (30.1%)	61 (32.6%)
70–79 years	169 (27.4%)	117 (26.3%)	47 (25.1%)
≥ 80 years	133 (21.6%)	76 (17.1%)	23 (12.3%)
Elderly patients			
≥ 65 years	393 (67.3%)	188 (42.2%)	87 (46.5%)
< 65 years	224 (36.3%)	257 (57.8%)	100 (53.5%)
Sex			
Female	346 (56.1%)	225 (50.6%)	87 (46.5%)
Male	271 (43.9%)	220 (49.4%)	100 (53.5%)
Years of diagnosis			
2000–2004	129 (20.9%)	97 (21.8%)	34 (18.2%)
2005–2010	206 (33.4%)	148 (33.3%)	64 (34.2%)
2011–2016	282 (45.7%)	200 (44.9%)	89 (47.6%)
ChT	238 (38.5%)	238 (53.5%)	88 (47.1%)
RT record	321 (52.0%)	321 (72.3%)	103 (55.1%)
RT sequence	*n* = 617	*n* = 445	*n* = 187
No RT AND/OR cancer-directed surgery	486 (78.8%)	314 (70.6%)	84 (44.9%)
Adjuvant	123 (19.9%)	123 (27.6%)	98 (53.4%)
Neoadjuvant	3 (0.5%)	3 (0.7%)	2 (1.1%)
Before AND after surgery	5 (0.8%)	5 (1.1%)	3 (1.6%)
Surgery	187 (30.3%)	187 (42%)	187 (100%)
Surgery type	*n* = 187	–	–
Lobectomy AND/OR isthmectomy	42 (22.4%)		
Removal of less than one lobe, NOS	34 (18.2%)		
Subtotal OR near-total thyroidectomy	22 (11.8%)		
Total thyroidectomy	89 (47.6%)		
Multimodal treatment	72 (11.7%)	72 (16.2%)	72 (38.5%)
Cause of death			
N/A not first tumor	94 (15.2%)	59 (13.3%)	31 (16.6%)
Dead, attributable to ATC	472 (76.5%)	346 (78.8%)	133 (71.1%)
Alive OR dead of other cause	44 (7.1%)	35 (7.9%)	21 (11.2%)
Dead, but COD missing/unknown	7 (1.1%)	5 (1.1%)	2 (1.1%)
Total number of patients	617	445	187

**Table 5:** Multivariate analysis of prognostic factors for OS and DSS of the SEER cohorts

Factor	Whole cohort						Without BSC					
	OS			DSS			OS			DSS		
	HR	95%CI	*p* value	HR	95%CI	*p* value	HR	95%CI	*p* value	HR	95%CI	*p* value
Surgery	1.953	1.557–2.450	** < 0.001**	1.803	1.275–2.550	** < 0.001**	1.769	1.289–2.427	** < 0.001**	1.913	1.286–2.845	0.001*
RT	1.880	1.566–2.258	** < 0.001**	1.611	1.191–2.178	0.002*	1.729	1.311–2.280	** < 0.001**	1.490	1.058–2.099	0.022*
ChT	1.723	1.409–2.108	** < 0.001**	1.572	1.197–2.064	0.001*	1.638	1.273–2.106	** < 0.001**	1.579	1.203–2.072	** < 0.001**
Multimodal treatment	0.692	0.478–1.002	0.051	0.900	0.542–1.494	0.683	0.790	0.499–1.249	0.313	0.844	0.504–1.413	0.518
Age ≥ 65 years	0.795	0.665–0.950	**0.012**	0.970	0.799–1.178	0.760	0.789	0.644–0.967	**0.022**	NA	NA	NA

Statistically significant values (*p*< 0.05) are in bold

**Fig. 4:**
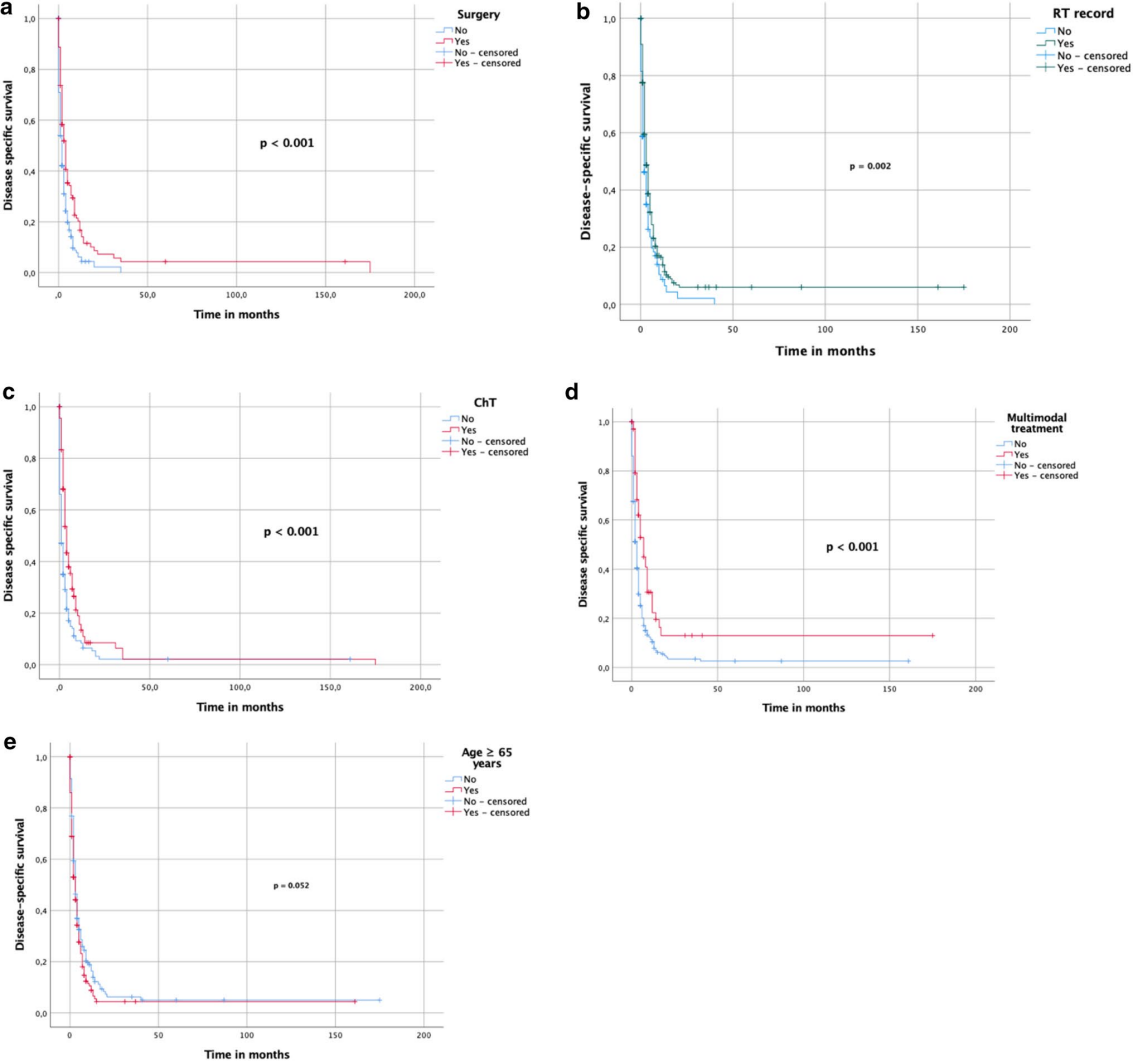
**A**–**E**. DSS of patients from SEER database without best supportive care only regimen

Furthermore, we eliminated 172 patients from our analysis, who did not receive any cancer-directed treatment, to verify predictors for an improved OS/DSS. On the univariate analysis surgery (*p* < 0.001), RT (*p* < 0.001), ChT (*p* < 0.001), multimodal treatment (*p* < 0.001), age ≥ 65 years (*p* < 0.001) and combinations of therapies (not trimodal) vs. RT alone (*p* < 0.001) or surgery alone (*p* < 0.001), correlated with an improved OS (Suppl. Figure 3A–E). Multivariately, surgery (HR 1.769, 95% CI 1.289–2.427, *p* < 0.001) administration of RT (HR 1.729, 95% CI 1.311–2.280, *p* < 0.001), ChT (HR 1.638, 95% CI 1.273–2.106, *p* < 0.001) and age <65 years (HR 0.789, 95% CI 0.644–0.967, *p* = 0.022) were independent predictors for an improved OS (Table 5). Univariate analyses for DSS showed similar correlations: surgery (*p* < 0.001), ChT (*p* < 0.001), RT (*p* = 0.002), multimodal treatment (*p* < 0.001), ChT without RT or without surgery vs. surgery and RT ± ChT (*p* < 0.001), combined therapies (not only trimodal) vs. RT only (*p* < 0.001) or surgery only (*p* = 0.008) (Fig. 4A–E). On the multivariate analysis surgery (HR 1.913, 95% CI 1.286–2.845, *p* = 0.001) administration of RT (HR 1.490, 95% CI 1.058–2.099, *p* = 0.022) and ChT (HR 1.579, 95% CI 1.203–2.072, *p* < 0.001) were independent predictors for an improved DSS (Table 5).

**Fig. 5:**
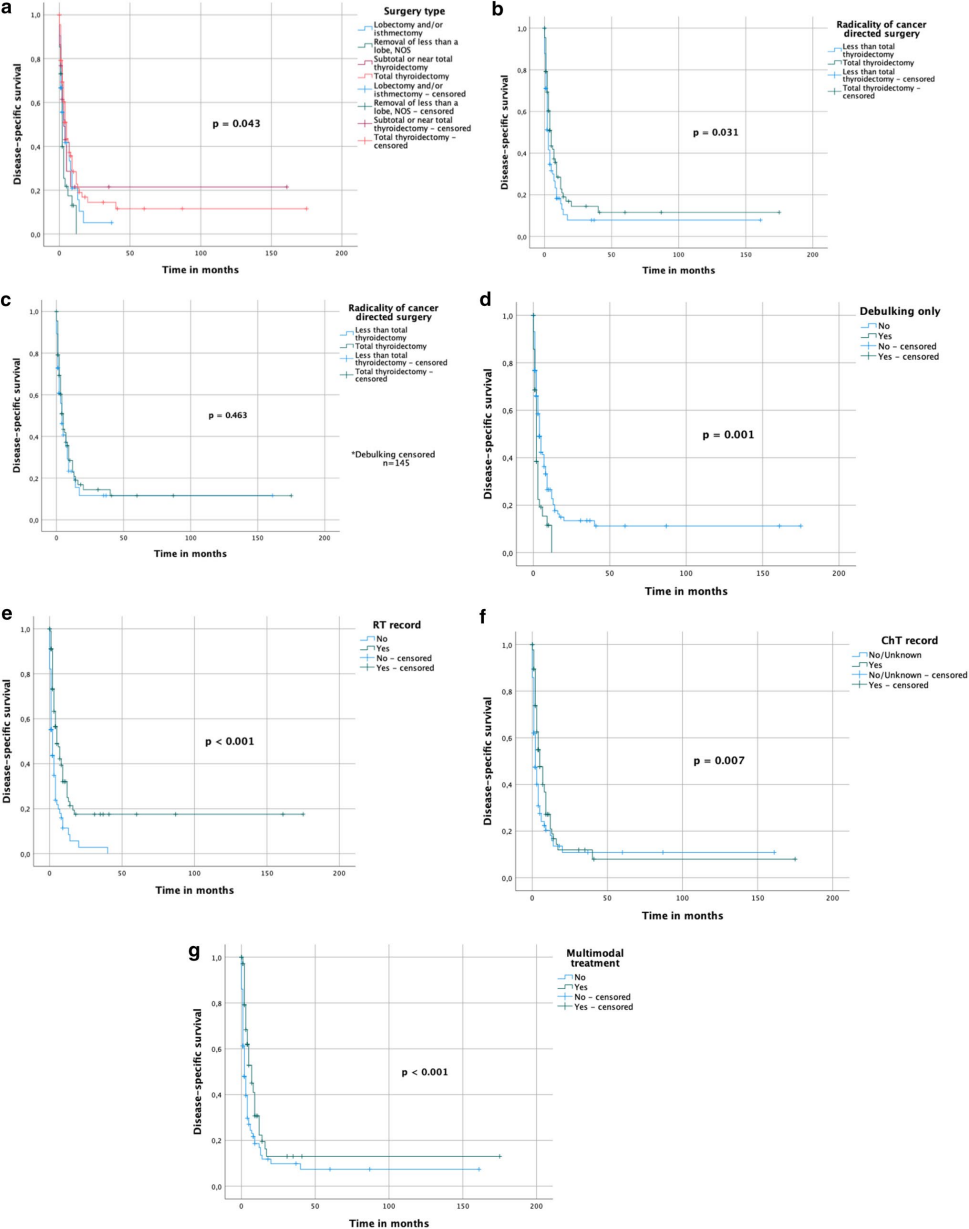
**A**–**G**. DSS of operated patients from SEER database

To identify specific surgical interventions, that correlate with best OS/DSS, we subsequently analyzed a separate cohort of patients, who all underwent surgery and/or any other cancer-directed treatment. On the univariate analysis, total thyroidectomy (*p* = 0.031), administration of ChT (*p* = 0.007), RT (*p* < 0.001), combination of surgery and RT ± ChT (*p* < 0.001) and multimodal treatment (*p* < 0.001) correlated with an improved DSS (Fig. 5A–I). Debulking surgery inversely correlated with the DSS (*p* < 0.001) (Fig. 5D). On the multivariate analysis, debulking surgery was an independent predictor for a worse outcome (HR 0.535, 95% CI 0.332–0.862, *p* = 0.010), whereas RT administration correlated with a longer DSS (HR 2.316, 95% CI 1.362–3.939, *p* = 0.002) (Table 6). Total, sub-total and near-total thyroidectomy showed significantly longer DSS than other thyroid surgeries (*p* = 0.043 and *p* = 0.031) (Fig. 5A, B). On the other hand, if debulking patients were eliminated from the analysis, there was no significant difference in the DSS, when comparing total and less than total thyroidectomy (OS–*p* = 0.115; DSS–*p* = 0.463, Fig. 5C, Suppl. Figure 4C). Similarly, total thyroidectomy (*p* = 0.005), administration of ChT (*p* < 0.001), RT (*p* < 0.001), as well as multimodality (*p* < 0.001) and age < 65 years (*p* = 0.002) correlated with a longer OS (Suppl. Figure 4A–I). However, on the multivariate analysis only administration of RT (HR 2.349, 95% CI 1.469–3.757, *p* < 0.001) and debulking (HR 1.564, 95% CI 1.024–2.389, *p* = 0.038) were independent predictors for OS (Table 6).

**Table 6:** Multivariate analysis of prognostic factors for OS and DSS within the operated SEER cohort

Factor	OS			DSS		
	HR	95%CI	*p* value	HR	95%CI	*p* value
Radicality of cancer-directed surgery	0.851	0.593–1.220	0.38	0.908	0.605–1.364	0.643
Debulking only	1.564	1.024–2.389	**0.038**	0.535	0.332–0.862	**0.010**
ChT administrated	1.433	0.818–2.511	0.209	1.150	0.651–2.033	0.630
RT administrated	2.349	1.469–3.757	** < 0.001**	2.316	1.362–3.939	**0.002**
Multimodal treatment	0.711	0.345–1.469	0.357	0.937	0.430–2.039	0.869
Age ≥ 65 years	0.794	0.570–1.105	0.171	NA	NA	NA

Statistically significant values (*p*< 0.05) are in bold

## Discussion

In the current study, we have evaluated the impact of different thyroid surgical strategies on OS and DSS in 3 different cohorts of patients with ATC stage IVC and found it to be associated with a significantly improved outcome. Furthermore, we showed that administration of RT, ChT and multimodal therapy, as well as younger age correlate with an improved OS or DSS.

The role of surgical treatment in metastatic ATC remains controversial. There are several treatment approaches at this stage, which may include surgery to the primary tumor and neck dissection depending on the specific guideline (Bible et al. 2021; Haddad et al. 2018; Filetti et al. 2019). Oncological surgical approaches in ATC vary from total thyroidectomy, subtotal or near total thyroidectomy to debulking. On the basis of tumor size and/or infiltration of adjacent structures, as well as extent of metastatic disease, i.e., T-/N-/M-stage, different strategies have to be considered within a multidisciplinary board. Removal of one of the lobes is less invasive, but yet only plausible in patients with intrathyroidal or incidental ATC, whereas total thyroidectomy is recommended by most of the guidelines (Bible et al. 2021; Haddad et al. 2018; Smallridge et al. 2012). If feasible and the tumor classified as resectable, R0/R1 resection has to be goal (Haddad et al. 2018), which, however, is rarely achievable given the infiltrative growth pattern (Filetti et al. 2019). Based on our systematic review, several studies have reported a negative resection margin to be associated with a survival benefit (Aslan et al. 2014; Glaser et al. 2016; Roche et al. 2010; Liu et al. 2016; Passler et al. 1999). However, our pooled analysis on the resection status did not show any correlation with survival, which may be attributed to a small and heterogeneous sample size of only 35 patients with accurate data on the resection margins. Since ATC is characterized by an aggressive growth, it often infiltrates neighboring structures, so that an option of an ultra-radical resection including laryngectomy, resection of the infrahyoid muscles, trachea or esophagus arises. Thus, Sugitani et al. (Sugitani et al. 2014) found ultra-radical surgery to offer a benefit for survival in patients with an ATC IVB. Conversely, Goffredo et al. (Goffredo et al. 2015) evaluated a retrospective cohort of 335 operated ATC patients and did not find a survival benefit for aggressive resections in stages IVB and IVC. In that study a missing potential benefit from surgery was attributed to the morbidities and operative risks of radical resections. In our systematic review we did not find any of the studies evaluating exclusively ATC IVC patients or comparing radical resections with limited thyroid surgeries. However, some large-scale studies (Glaser et al. 2016; Sugitani et al. 2012; Gui et al. 2020; Ridder et al. 2020; Haymart et al. 2013; Kebebew et al. 2005; Huang et al. 2019; Pezzi et al. 2017) have reported their cohorts to contain up to 56.1% stage IVC patients and all of these studies found surgery to be associated with an improved OS (Gui et al. 2020). This is similar to our findings, that surgical treatment to the primary tumor also in an advanced stage ATC is an appropriate treatment option and can bear a significant survival benefit in selected patients. In addition, we have shown total thyroidectomy as a specified surgical approach to correlate with an improved OS/DSS. In that analysis we found no difference between TT and less than TT surgeries excluding debulking, i.e., compared subtotal-, near total thyroidectomies or lobectomies. This is in line with a study of Venkatesh et al. who reported less invasive thyroidectomies to be non-inferior to TT in terms of OS (Venkatesh et al. 1990). In our pooled analysis, we were only able to see a tendency regimen toward an improved OS for TT (*p* = 0.058).

Removal of the gross tumor in the head and neck region is of crucial importance for adjuvant RT or ChT, since it facilitates a beneficial local control by these therapies, as it was shown for ATC by some authors (Glaser et al. 2016; Fan et al. 2020). However, the extent of primary tumor-directed surgery needs to take into consideration the extent of primary tumor spread. The risk–benefit-ratio needs to be thoroughly evaluated between surgery to the primary tumor which may enhance therapeutic outcome and the risk of surgery-induced morbidity and delay of systemic therapy. The main goal of surgery to the primary tumor in systemically metastasized ATC is to avoid potential complications from the locally destructive tumor growth leading to obstructions of airway and hence respiratory insufficiency, as well as compression and/or infiltration of carotid vessels, as these are common death causes reported to date (Kitamura et al. 1999). Nilsson et al. investigated debulking surgery to the primary tumor in ATC and found it to improve patients’ outcome within multimodality approach (Nilsson et al. 1998). Debulking as palliative surgery in ATC IVC is, however, generally not recommended, as there is no sufficient evidence for a patient’s benefit within the multimodality approach, where urgently necessary systemic treatment is of highest priority(Bible et al. 2021; Haddad et al. 2018; Filetti et al. 2019; Sugitani et al. 2018). In our analyses debulking surgery was an independent predictor for a higher overall and disease-specific mortality in ATC IVC patients. This may be caused by the unfavorable constellation of peri-operative morbidity and insufficient response towards RT or systemic therapies in terms of local control. Moreover, debulking surgery is only performed in cases, where R0/R1 resection is not achievable in locally aggressive advanced disease, which is a prognostically unfavorable constellation by itself. In addition, debulking surgery postpones the start of RT and/or systemic therapy and can lead to intervention-related complications with a negative impact on the outcome.

In general, there are several specific complications after an oncologic thyroid surgery: permanent and transient uni- or bilateral recurrent laryngeal nerve palsy, injuries to the superior laryngeal nerve, tracheomalacia, hypoparathyroidism, and fistulae (Rosato et al. 2004; Oertli and Udelsman 2007). The incidence of nerve injury, the most common specific complication, has been sufficiently decreased by the utilization of an intraoperative neurological monitoring, as suggested by some authors (Bai and Chen 2018; Zheng et al. 2013). Other complications can effectively be managed with either conservative or additional invasive approaches (Orloff et al. 2018; Campisi et al. 2013; Lee et al. 2016a; Spitzweg et al. 2017). These potential risks in the course of surgery, especially debulking, seem to outweigh the profit from this intervention because of the heterogeneous volume of the remaining tumor burden. Thus, limited (not ultra-radical) thyroidectomy, but not debulking, may be offered in selected patients with ATC IVC, since there is a promising evidence of potential profit, whereas complications can appropriately be avoided or managed. Furthermore, data suggest that at least < R2 resection has to be achieved to facilitate further therapeutic response.

In ATC, surgery is recommended to be followed by adjuvant therapy, consisting of local RT with or without simultaneous or sequential ChT, to reduce the risk of local recurrence and thus improve overall survival. The recommended radiation doses range between 20 and 75 Gy, depending on the therapeutic goal (Filetti et al. 2019; Pezzi et al. 2017). For palliative radiation, doses between 20 and 30 Gy are usually administered; for a curative therapeutic goal, doses of ≥ 40 Gy are used (Liu et al. 2016; Sugitani et al. 2018; Sun et al. 2015; Wendler et al. 2016). However, there is still disagreement about the level of doses administered to patients with a curative therapy goal. In addition to studies recommending ≥ 40 Gy for ATC patients, however, many indicate effective irradiation only at doses of ≥ 60 Gy. According to a study by Fan et al. for example, irradiation doses of ≥ 60 Gy improve overall survival (*p* = 0.004), as well as local control (*p* < 0.001) and additionally prolong median overall survival (10.6 months vs. 3.6 months)(Fan et al. 2020). Similar results were obtained in the study by Glaser et al. which indicates an effective radiation dose for a favorable outcome at ≥ 59.4 Gy (Glaser et al. 2016). A more aggressive therapy regimen with higher radiation doses not only shows a benefit in stage IVA, but also in selected patients in inoperable stage IVB or stage IVC. Higher doses have a positive effect on local control and reduce the risk of local recurrence and thus improve overall survival rates (Pezzi et al. 2017). The evaluations of Fan et al. also suggest that higher radiation doses do not necessarily mean higher toxicity and that grade 4 toxicities did not occur more frequently than with lower radiation doses (Fan et al. 2020). In our pooled analysis from the systematic review, sufficient radiation doses beyond 30 Gy correlated uni- and multivariately with a beneficial OS (Table 3, Fig. 2C). Analyses obtained from the SEER database also show RT to be an independent predictor for a beneficial OS and DSS (Suppl. Figures 1C, 2B); however, exact dosage remains unknown due to limited SEER data. Furthermore, our previous study showed that RT in advanced ATC may also offer a durable local control and can be considered safe for patients (Augustin et al. 2021).

The application of ChT, usually in combination with adjuvant RT, is still controversial. Systemic therapy is the main treatment regimen for metastatic patients, but has only a low response rate and usually leads to significant side effects with a corresponding loss of quality of life (Filetti et al. 2019). There are studies that show that ChT in ATC does not bring a survival benefit and only increases therapy-associated toxicities (Huang et al. 2019; Sun et al. 2015; Corrigan et al. 2019). Other studies, however, found a survival benefit that can be achieved by simultaneously or sequentially administered ChT (Wendler et al. 2016; Käsmann et al. 2016). Recommended ChT regimens include either single-agent therapy with paclitaxel or doxorubicin, or a combination of agents, such as carboplatin/paclitaxel and docetaxel/doxorubicin (Haddad et al. 2018; Filetti et al. 2019; Ain KB et al. (CATCHIT) Group* 2000; ; ; Sosa et al. 2014; Shimaoka et al. 1985). In our analysis, administration of ChT corresponded with beneficial OS and DSS rates in univariate analysis and also in multivariate analysis in both SEER cohorts (Suppl. Figures 1B, C, 3C, F, 4F; Fig. 4C; 5F; Tables 5 and 6), but also univariately in the pooled cohort (Fig. 2B).

Combination of all three therapeutic approaches in the course of a multimodality therapy approach shows an advantage in the vast majority of patients with regard to overall survival and progression-free survival(Haymart et al. 2013; Pezzi et al. 2017; Fan et al. 2020; Corrigan et al. 2019; Rao et al. 2017). In stage IVA and resectable stage IVB, this approach is already an established standard of care (Haddad et al. 2018; Filetti et al. 2019; Smallridge et al. 2012; Sun et al. 2015). However, some studies are extending multimodal therapy to patients in stage IVB and stage IVC. Tian et al. showed CRT in ATC patients with metastatic disease to correlate with a longer 1-year OS (HR 0.65, *p* < 0.001) (Tian et al. 2020). Depending on the physical condition of the patients and their personal expectations, a more intensive therapy regime should, therefore, be considered. The decision on the individual therapy approach should be made within the framework of an interdisciplinary expert team of oncologists, radiotherapists, endocrinologists, pathologists and surgeons. In our analyses, multimodal therapy in ATC IVC was associated with a prolonged OS and DSS in all of our cohorts on the univariate analysis (*p* value: < 0.001–0.014, Figs. 2–5, Suppl. Figures 1–4). On the multivariate analysis, it, however, did not reach any significance (Fig. 5G, Suppl. Figure 4G).

In the course of our investigation, we also evaluated the impact of older age ≥ 65 years in metastatic ATC on the OS and DSS. We found it to be an independent predictor for a higher overall mortality in both SEER databases, but not for the disease-specific mortality (Table 5, Suppl. Figures 1F, 2E, 3E, 4I; Fig. 4E). This is in line with the findings of other authors that stated age ≥ 65 and ≥ 70 years, respectively, to be an independent predictor for a shortened OS (Glaser et al. 2016; Sugitani et al. 2012; Pezzi et al. 2017). Such a difference is most likely caused by the lower performance score and lower susceptibility for intensive treatment regimens in older patients.

In addition, some authors reported a significant increase in survival of ATC patients within the last two decades due to a significantly improved patient-management (Maniakas et al. 2020). Prasongsook et al. have also shown a difference between treatment outcome in ATC patients, yet not for the multimodal approach in the metastasized ATC (Prasongsook et al. 2017). In none of our analysis, however, we were able to find any difference in the patient outcome depending on the year of their diagnosis. Conversely, we have only investigated an advanced stage ATC. These findings, however, were generated from the data of an era prior to Food and Drug Administration approvals (FDA, USA) of any of the available TKIs or immunotherapies for ATC. A combination of dabrafenib and trametinib has been approved by FDA for an advanced, *BRAF V600E/MEK* positive ATC, as it showed acceptable toxicity with an overall response rate (ORR) of 69% in May 2018 (Subbiah et al. 2018; Highlights of prescribing information xxxx). Furthermore, first results of the phase-II ATLEP trial on the combination of Lenvatinib and pembrolizumab in the metastasized ATC/PDTC showed a promising outcome with acceptable toxicity(Dierks et al. 2021). In total, not only may these therapies provide an improved survival, distant and local control rates, but it may also facilitate a reevaluation of the role of surgery in ATC stage IVC, especially when used in a neo-adjuvant setting.

Our study has several limitations, such as the retrospective nature and, hence, the risk of including hidden selection biases. In addition, SEER data on ChT and RT have been reported to have limitations in terms of sensitivity, biases and variables within the treatment sequence (Noone et al. 2016). The SEER data have, however, still a significant positive-predictive value, but have to be used with caution. In our analysis, we partially investigated patients with any tumor-directed treatment and did not evaluate any treatment-related sequences, to minimize possible biases between treated and untreated patients.

In conclusion, we were able to show that surgery to the primary tumor—thyroidectomy in any form, but not debulking—was an important factor bearing an OS and a DSS benefit for ATC patients with distant metastases from SEER database. Furthermore, sufficient RT ( ≥ 30 Gy), administration of ChT or combined multimodal treatment and young age < 65 years had a significant influence on the OS and DSS from both pooled and SEER-based analysis.

## Supplementary Information

Below is the link to the electronic supplementary material.

Supplementary file1 (DOCX 31016 KB)

## References

[CR1] Ain KB, Egorin MJ, DeSimone PA, Collaborative Anaplastic Thyroid Cancer Health Intervention Trials (CATCHIT) Group (2000). Treatment of anaplastic thyroid carcinoma with paclitaxel: phase 2 trial using ninety-six-hour infusion. Thyroid.

[CR2] Akaishi J, Sugino K, Kitagawa W, Nagahama M, Kameyama K, Shimizu K, Ito K, Ito K (2011). Prognostic factors and treatment outcomes of 100 cases of anaplastic thyroid carcinoma. Thyroid.

[CR3] Aslan ZAT, Granados-García M, Luna-Ortiz K, Guerrero-Huerta FJ, Gómez-Pedraza A, Ñamendys-Silva SA, Meneses-García A, Ordoñez-Mosquera JM (2014) Anaplastic thyroid cancer: multimodal treatment results. 13 10.3332/ecancer.2014.449 PMC4118732 25114721

[CR4] Augustin T, Oliinyk D, Rauch J, Koehler VF, Spitzweg C, Belka C, Käsmann L (2021). Radiation to the primary tumor in metastatic anaplastic thyroid cancer. In Vivo.

[CR5] Baek S-K, Lee M-C, Hah JH, Ahn S-H, Son Y-I, Rho Y-S, Chung P-S, Lee Y-S, Koo BS, Jung K-Y, Lee B-J (2017). Role of surgery in the management of anaplastic thyroid carcinoma: Korean nationwide multicenter study of 329 patients with anaplastic thyroid carcinoma, 2000 to 2012: surgical role in anaplastic thyroid carcinoma. Head Neck.

[CR6] Bai B, Chen W (2018). Protective effects of intraoperative nerve monitoring (IONM) for recurrent laryngeal nerve injury in thyroidectomy: meta-analysis. Sci Rep.

[CR7] Bhatia A, Rao A, Ang K-K, Garden AS, Morrison WH, Rosenthal DI, Evans DB, Clayman G, Sherman SI, Schwartz DL (2009). Anaplastic thyroid cancer: clinical outcomes with conformal radiotherapy. Head Neck NA-NA.

[CR8] Bible KC, Kebebew E, Brierley J, Brito JP, Cabanillas ME, Clark TJ, Di Cristofano A, Foote R, Giordano T, Kasperbauer J, Newbold K, Nikiforov YE, Randolph G, Rosenthal MS, Sawka AM, Shah M, Shaha A, Smallridge R, Wong-Clark CK (2021). 2021 American thyroid association guidelines for management of patients with anaplastic thyroid cancer: American thyroid association anaplastic thyroid cancer guidelines task force. Thyroid.

[CR9] Brignardello E, Gallo M, Baldi I, Palestini N, Piovesan A, Grossi E, Ciccone G, Boccuzzi G (2007). Anaplastic thyroid carcinoma: clinical outcome of 30 consecutive patients referred to a single institution in the past 5 years. Eur J Endocrinol.

[CR10] Brignardello E, Palestini N, Felicetti F, Castiglione A, Piovesan A, Gallo M, Freddi M, Ricardi U, Gasparri G, Ciccone G, Arvat E, Boccuzzi G (2014). Early surgery and survival of patients with anaplastic thyroid carcinoma: analysis of a case series referred to a single institution between 1999 and 2012. Thyroid.

[CR11] Busnardo B, Daniele O, Pelizzo MR, Mazzarotto R, Nacamulli D, DeVido D, Mian C, Girelli ME (2000). A multimodality therapeutic approach in anaplastic thyroid carcinoma: study on 39 patients. J Endocrinol Invest.

[CR12] Campisi CC, Boccardo F, Piazza C, Campisi C (2013). Evolution of chylous fistula management after neck dissection. Curr Opin Otolaryngol Head Neck Surg.

[CR13] Corrigan KL, Williamson H, Elliott Range D, Niedzwiecki D, Brizel DM, Mowery YM (2019). Treatment outcomes in anaplastic thyroid cancer. J Thyroid Res.

[CR14] Dal Maso L, Tavilla A, Pacini F, Serraino D, van Dijk BAC, Chirlaque MD, Capocaccia R, Larrañaga N, Colonna M, Agius D, Ardanaz E, Rubió-Casadevall J, Kowalska A, Virdone S, Mallone S, Amash H, De Angelis R, Hackl M, Zielonke N, Van Eycken E, Henau K, Valerianova Z, Dimitrova N, Sekerija M, Dušek L, Zvolský M, Storm H, Engholm G, Mägi M, Aareleid T, Malila N, Seppä K, Velten M, Guizard AV, Faivre J, Woronoff AS, Tretarre B, Bossard N, Uhry Z, Colonna M, Molinié F, Bara S, Schvartz C, Lapôtre-Ledoux B, Grosclaude P, Stabenow R, Luttmann S, Eberle A, Brenner H, Nennecke A, Engel J, Schubert-Fritschle G, Heidrich J, Holleczek B, Katalinic A, Jónasson JG, Tryggvadóttir L, Comber H, Mazzoleni G, Bulatko A, Buzzoni C, Giacomin A, Sutera Sardo A, Mazzei A, Ferretti S, Barchielli A, Caldarella A, Gatta G, Sant M, Amash H, Amati C, Baili P, Berrino F, Bonfarnuzzo S, Botta L, Capocaccia R, Di Salvo F, Foschi R, Margutti C, Meneghini E, Minicozzi P, Trama A, Serraino D, Zucchetto A, De Angelis R, Caldora M, Carrani E, Francisci S, Mallone S, Pierannunzio D, Roazzi P, Rossi S, Santaquilani M, Tavilla A, Pannozzo F, Busco S, Filiberti RA, Vercelli M, Ricci P, Autelitano M, Spagnoli G, Cirilli C, Fusco M, Vitale MF, Usala M, Vitale F, Ravazzolo B, Michiara M, Tumino R, Mangone L, Vicentini M, Falcini F, Iannelli A, Sechi O, Cesaraccio R, Piffer S, Madeddu A, Tisano F, Maspero S, Fanetti AC, Zanetti R, Rosso S, Candela P, Scuderi T, Stracci F, Rocca A, Tagliabue G, Contiero P, Rugge M, Tognazzo S, Pildava S, Smailyte G, Calleja N, Agius D, Johannesen TB, Rachtan J, Góźdź S, Mężyk R, Błaszczyk J, Bębenek M, Bielska-Lasota M, Forjaz de Lacerda G, Bento MJ, Castro C, Miranda A, Mayer-da-Silva A, Safaei Diba C, Primic-Zakelj M, Errezola M, Bidaurrazaga J, Díaz García JM, Marcos-Navarro AI, Marcos-Gragera R, Izquierdo Font A, Sanchez MJ, Molina E, Navarro C, Chirlaque MD, Moreno-Iribas C, Ardanaz E, Galceran J, Carulla M, Lambe M, Khan S, Mousavi M, Bouchardy C, Usel M, Ess SM, Frick H, Lorez M, Ess SM, Herrmann C, Bordoni A, Spitale A, Konzelmann I, Visser O, Ho V, Otter R, Coleman M, Allemani C, Rachet B, Rashbass J, Broggio J, Verne J, Gavin A, Donnelly C, Brewster DH, Huws DW, White C (2017). Survival of 86,690 patients with thyroid cancer: a population-based study in 29 European countries from EUROCARE-5. Eur J Cancer.

[CR15] De Crevoisier R, Baudin E, Bachelot A, Leboulleux S, Travagli J-P, Caillou B, Schlumberger M (2004). Combined treatment of anaplastic thyroid carcinoma with surgery, chemotherapy, and hyperfractionated accelerated external radiotherapy. Int J Radiat Oncol.

[CR16] de Ridder M, Nieveen van Dijkum E, Engelsman A, Kapiteijn E, Klümpen H-J, Rasch CRN (2020). Anaplastic thyroid carcinoma: a nationwide cohort study on incidence, treatment and survival in the Netherlands over 3 decades. Eur J Endocrinol.

[CR17] Derbel O, Limem S, Ségura-Ferlay C, Lifante J-C, Carrie C, Peix J-L, Borson-Chazot F, Bournaud C, Droz J-P, de la Fouchardière C (2011). Results of combined treatment of anaplastic thyroid carcinoma (ATC). BMC Cancer.

[CR18] Dierks C, Seufert J, Aumann K, Ruf J, Klein C, Kiefer S, Rassner M, Boerries M, Zielke A, la Rosee P, Meyer PT, Kroiss M, Weißenberger C, Schumacher T, Metzger P, Weiss H, Smaxwil C, Laubner K, Duyster J, von Bubnoff N, Miething C, Thomusch O (2021). Combination of lenvatinib and pembrolizumab is an effective treatment option for anaplastic and poorly differentiated thyroid carcinoma. Thyroid.

[CR19] Dumke A-K, Pelz T, Vordermark D (2014). Long-term results of radiotherapy in anaplastic thyroid cancer. Radiat Oncol.

[CR20] Fan D, Ma J, Bell AC, Groen AH, Olsen KS, Lok BH, Leeman JE, Anderson E, Riaz N, McBride S, Ganly I, Shaha AR, Sherman EJ, Tsai CJ, Kang JJ, Lee NY (2020). Outcomes of multimodal therapy in a large series of patients with anaplastic thyroid cancer. Cancer.

[CR21] Filetti S, Durante C, Hartl D, Leboulleux S, Locati LD, Newbold K, Papotti MG, Berruti A (2019). Thyroid cancer: ESMO clinical practice guidelines for diagnosis, treatment and follow-up. Ann Oncol.

[CR22] Glaser SM, Mandish SF, Gill BS, Balasubramani GK, Clump DA, Beriwal S (2016). Anaplastic thyroid cancer: prognostic factors, patterns of care, and overall survival: anaplastic thyroid cancer. Head Neck.

[CR23] Goffredo P, Thomas SM, Adam MA, Sosa JA, Roman SA (2015). Impact of timeliness of resection and thyroidectomy margin status on survival for patients with anaplastic thyroid cancer: an analysis of 335 cases. Ann Surg Oncol.

[CR24] Gui W, Zhu W, Lu W, Shang C, Zheng F, Lin X, Li H (2020). Development and validation of a prognostic nomogram to predict overall survival and cancer-specific survival for patients with anaplastic thyroid carcinoma. PeerJ.

[CR25] Haddad RI, Nasr C, Bischoff L, Busaidy NL, Byrd D, Callender G, Dickson P, Duh Q-Y, Ehya H, Goldner W, Haymart M, Hoh C, Hunt JP, Iagaru A, Kandeel F, Kopp P, Lamonica DM, McIver B, Raeburn CD, Ridge JA, Ringel MD, Scheri RP, Shah JP, Sippel R, Smallridge RC, Sturgeon C, Wang TN, Wirth LJ, Wong RJ, Johnson-Chilla A, Hoffmann KG, Gurski LA (2018). NCCN guidelines insights: thyroid carcinoma, version 2.2018. J Natl Compr Canc Netw.

[CR26] Haigh PI, Ituarte PHG, Wu HS, Treseler PA, Posner MD, Quivey JM, Duh QY, Clark OH (2001). Completely resected anaplastic thyroid carcinoma combined with adjuvant chemotherapy and irradiation is associated with prolonged survival. Cancer.

[CR27] Haigh PI, Ituarte PHG, Wu HS, Treseler PA, Posner MD, Quivey JM, Duh QY, Clark OH (2001). Completely resected anaplastic thyroid carcinoma combined with adjuvant chemotherapy and irradiation is associated with prolonged survival. Cancer.

[CR28] Haymart MR, Banerjee M, Yin H, Worden F, Griggs JJ (2013). Marginal treatment benefit in anaplastic thyroid cancer: treatment of anaplastic thyroid cancer. Cancer.

[CR29] Highlights of prescribing information. https://www.accessdata.fda.gov/drugsatfda_docs/label/2018/202806s010lbl.pdf

[CR30] Huang N, Shi X, Lei B, Wei W, Lu Z, Yu P, Wang Y, Ji Q, Wang Y (2019). An update of the appropriate treatment strategies in anaplastic thyroid cancer: a population-based study of 735 patients. Int J Endocrinol.

[CR31] Ito K, Hanamura T, Murayama K, Okada T, Watanabe T, Harada M, Ito T, Koyama H, Kanai T, Maeno K, Mochizuki Y, Amano J (2012). Multimodality therapeutic outcomes In anaplastic thyroid carcinoma: improved survival in subgroups of patients with localized primary tumors. Head Neck.

[CR32] Jacobsen A-B, Grøholt KK, Lorntzsen B, Osnes TA, Falk RS, Sigstad E (2017). Anaplastic thyroid cancer and hyperfractionated accelerated radiotherapy (HART) with and without surgery. Eur Arch Otorhinolaryngol.

[CR33] Käsmann L, Janssen S, Rades D (2016). Karnofsky performance score, radiation dose and nodal status predict survival of elderly patients irradiated for limited-disease small-cell lung cancer. Anticancer Res.

[CR34] Kebebew E, Greenspan FS, Clark OH, Woeber KA, McMillan A (2005). Anaplastic thyroid carcinoma: treatment outcome and prognostic factors. Cancer.

[CR35] Kihara M, Miyauchi A, Yamauchi A, Yokomise H (2004). Prognostic factors of anaplastic thyroid carcinoma. Surg Today.

[CR36] Kim TY, Kim KW, Jung TS, Kim JM, Kim SW, Chung K-W, Kim EY, Gong G, Oh YL, Cho SY, Yi KH, Kim WB, Park DJ, Chung JH, Cho BY, Shong YK (2007). Prognostic factors for Korean patients with anaplastic thyroid carcinoma. Head Neck.

[CR37] Kitamura Y, Shimizu K, Nagahama M, Sugino K, Ozaki O, Mimura T, Ito K, Ito K, Tanaka S (1999). Immediate causes of death in thyroid carcinoma: clinicopathological analysis of 161 fatal cases. J Clin Endocrinol Metabol.

[CR38] Lee DY, Won J-K, Lee S-H, Park DJ, Jung KC, Sung M-W, Wu H-G, Kim KH, Park YJ, Hah JH (2016). Changes of clinicopathologic characteristics and survival outcomes of anaplastic and poorly differentiated thyroid carcinoma. Thyroid.

[CR39] Lee DY, Won J-K, Choi HS, Park DJ, Jung KC, Sung M-W, Kim KH, Hah JH, Park YJ (2016). recurrence and survival after gross total removal of resectable undifferentiated or poorly differentiated thyroid carcinoma. Thyroid.

[CR40] Lim SM, Shin S-J, Chung WY, Park CS, Nam K-H, Kang S-W, Keum KC, Kim JH, Cho JY, Hong YK, Cho BC (2012). Treatment outcome of patients with anaplastic thyroid cancer: a single center experience. Yonsei Med J.

[CR41] Liu T-R, Xiao Z-W, Xu H-N, Long Z, Wei F-Q, Zhuang S-M, Sun X-M, Xie L-E, Mu J-S, Yang A-K, Zhang G-P, Fan Y (2016). Treatment and prognosis of anaplastic thyroid carcinoma: a clinical study of 50 cases. PLoS ONE.

[CR42] Maniakas A, Dadu R, Busaidy NL, Wang JR, Ferrarotto R, Lu C, Williams MD, Gunn GB, Hofmann M-C, Cote G, Sperling J, Gross ND, Sturgis EM, Goepfert RP, Lai SY, Cabanillas ME, Zafereo M (2020). Evaluation of overall survival in patients with anaplastic thyroid carcinoma, 2000–2019. JAMA Oncol.

[CR43] McIver B, Hay ID, Giuffrida DF, Dvorak CE, Grant CS, Thompson GB, van Heerden JA, Goellner JR (2001). Anaplastic thyroid carcinoma: a 50-year experience at a single institution. Surgery.

[CR44] Mohebati A, DiLorenzo M, Palmer F, Patel SG, Pfister D, Lee N, Tuttle RM, Shaha AR, Shah JP, Ganly I (2014). Anaplastic thyroid carcinoma: a 25-year single-institution experience. Ann Surg Oncol.

[CR45] Neff RL, Farrar WB, Kloos RT, Burman KD (2008). Anaplastic thyroid cancer. Endocrinol Metab Clin North Am.

[CR46] Nilsson O, Lindeberg J, Zedenius J, Ekman E, Tennvall J, Blomgren H, Grimelius L, Lundell G, Wallin G (1998). Anaplastic giant cell carcinoma of the thyroid gland: treatment and survival over a 25-year period. World J Surg.

[CR47] Noone A-M, Lund JL, Mariotto A, Cronin K, McNeel T, Deapen D, Warren JL (2016). Comparison of SEER treatment data with medicare claims. Med Care.

[CR48] Oertli D, Udelsman R (2007). Surgery of the thyroid and parathyroid glands.

[CR49] Orloff LA, Wiseman SM, Bernet VJ, Fahey TJ, Shaha AR, Shindo ML, Snyder SK, Stack BC, Sunwoo JB, Wang MB, For the American Thyroid Association Surgical Affairs Committee Writing Task Force (2018). American thyroid association statement on postoperative hypoparathyroidism: diagnosis, prevention, and management in adults. Thyroid.

[CR50] Passler C, Scheuba C, Prager G, Kaserer K, Flores JA, Vierhapper H, Niederle B (1999). Anaplastic (undifferentiated) thyroid carcinoma (ATC). Langenbecks Arch Surg.

[CR51] Paunovic IR, Sipetic SB, Zoric GV, Diklic AD, Savic DV, Marinkovic J, Zivaljevic VR (2015). Survival and prognostic factors of anaplastic thyroid carcinoma. Acta Chir Belg.

[CR52] Pezzi TA, Mohamed ASR, Sheu T, Blanchard P, Sandulache VC, Lai SY, Cabanillas ME, Williams MD, Pezzi CM, Lu C, Garden AS, Morrison WH, Rosenthal DI, Fuller CD, Gunn GB (2017). Radiation therapy dose is associated with improved survival for unresected anaplastic thyroid carcinoma: outcomes from the national cancer data base: unresected anaplastic thyroid carcinoma. Cancer.

[CR53] Pierie J-PEN, Muzikansky A, Gaz RD, Faquin WC, Ott MJ (2002). The effect of surgery and radiotherapy on outcome of anaplastic thyroid carcinoma. Ann Surg Oncol.

[CR54] Prasongsook N, Kumar A, Chintakuntlawar AV, Foote RL, Kasperbauer J, Molina J, Garces Y, Ma D, Wittich MAN, Rubin J, Richardson R, Morris J, Hay I, Fatourechi V, McIver B, Ryder M, Thompson G, Grant C, Richards M, Sebo TJ, Rivera M, Suman V, Jenkins SM, Smallridge RC, Bible KC (2017). Survival in response to multimodal therapy in anaplastic thyroid cancer. J Clin Endocrinol Metab.

[CR55] Rao SN, Zafereo M, Dadu R, Busaidy NL, Hess K, Cote GJ, Williams MD, William WN, Sandulache V, Gross N, Gunn GB, Lu C, Ferrarotto R, Lai SY, Cabanillas ME (2017). Patterns of treatment failure in anaplastic thyroid carcinoma. Thyroid.

[CR56] Roche B, Larroumets G, Dejax C, Kwiatkowsi F, Desbiez F, Thieblot P, Tauveron I (2010). Epidemiology, clinical presentation, treatment and prognosis of a regional series of 26 anaplastic thyroid carcinomas (ATC) Comparison with the Literature. Ann Endocrinol.

[CR57] Rosato L, Avenia N, Bernante P, De Palma M, Gulino G, Nasi PG, Pelizzo MR, Pezzullo L (2004). Complications of thyroid surgery: analysis of a multicentric study on 14,934 patients operated on in Italy over 5 years. World J Surg.

[CR58] Shimaoka K, Schoenfeld DA, Dewys WD, Creech RH, Deconti R (1985). A randomized trial of doxorubicin versus doxorubicin plus cisplatin in patients with advanced thyroid carcinoma. Cancer.

[CR59] Smallridge RC, Ain KB, Asa SL, Bible KC, Brierley JD, Burman KD, Kebebew E, Lee NY, Nikiforov YE, Rosenthal MS, Shah MH, Shaha AR, Tuttle for the American Thyroid Ass RM (2012). American Thyroid Association Guidelines for Management of Patients with Anaplastic Thyroid Cancer. Thyroid.

[CR60] So K, Smith RE, Davis SR (2017). Radiotherapy in anaplastic thyroid carcinoma: an Australian experience. J Med Imaging Radiat Oncol.

[CR61] Sosa JA, Elisei R, Jarzab B, Balkissoon J, Lu S, Bal C, Marur S, Gramza A, Yosef RB, Gitlitz B, Haugen BR, Ondrey F, Lu C, Karandikar SM, Khuri F, Licitra L, Remick SC (2014). Randomized safety and efficacy study of fosbretabulin with paclitaxel/carboplatin against anaplastic thyroid carcinoma. Thyroid.

[CR62] Spitzweg C, Reincke M, Gärtner R (2017). Schilddrüsennotfälle: thyreotoxische krise und myxödemkoma. Internist.

[CR63] Stavas MJ, Shinohara ET, Attia A, Ning MS, Friedman JM, Cmelak AJ (2014). Short course high dose radiotherapy in the treatment of anaplastic thyroid carcinoma. J Thyroid Res.

[CR64] Subbiah V, Kreitman RJ, Wainberg ZA, Cho JY, Schellens JHM, Soria JC, Wen PY, Zielinski C, Cabanillas ME, Urbanowitz G, Mookerjee B, Wang D, Rangwala F, Keam B (2018). Dabrafenib and trametinib treatment in patients with locally advanced or metastatic *BRAF* v600–mutant anaplastic thyroid cancer. J Clin Oncol.

[CR65] Sugitani I, Miyauchi A, Sugino K, Okamoto T, Yoshida A, Suzuki S (2012). Prognostic factors and treatment outcomes for anaplastic thyroid carcinoma: ATC research consortium of japan cohort study of 677 patients. World J Surg.

[CR66] Sugitani I, Hasegawa Y, Sugasawa M, Tori M, Higashiyama T, Miyazaki M, Hosoi H, Orita Y, Kitano H (2014). Super-radical surgery for anaplastic thyroid carcinoma: a large cohort study using the anaplastic thyroid carcinoma research consortium of Japan database: Super-radical surgery for anaplastic thyroid carcinoma. Head Neck.

[CR67] Sugitani I, Onoda N, Ito K, Suzuki S (2018). Management of anaplastic thyroid carcinoma: the fruits from the ATC research consortium of Japan. J Nippon Med Sch.

[CR68] Sun C, Li C, Hu Z, Li X, He J, Song M, Li G, Zhang F, Li Q (2015). Influence of risk grouping on therapeutic decisions in patients with anaplastic thyroid carcinoma. Eur Arch Otorhinolaryngol.

[CR69] Takahashi N, Matsushita H, Umezawa R, Yamamoto T, Ishikawa Y, Katagiri Y, Tasaka S, Takeda K, Fukui K, Kadoya N, Ito K, Jingu K (2019). Hypofractionated radiotherapy for anaplastic thyroid carcinoma: 15 years of experience in a single institution. Eur Thyroid J.

[CR70] Tennvall J, Lundell G, Wahlberg P, Bergenfelz A, Grimelius L, Åkerman M, Hjelm Skog A-L, Wallin G (2002). Anaplastic thyroid carcinoma: three protocols combining doxorubicin, hyperfractionated radiotherapy and surgery. Br J Cancer.

[CR71] Tian S, Switchenko JM, Fei T, Press RH, Abugideiri M, Saba NF, Owonikoko TK, Chen AY, Beitler JJ, Curran WJ, Gillespie TW, Higgins KA (2020). Survival advantage of chemoradiotherapy in anaplastic thyroid carcinoma: propensity score matched analysis with multiple subgroups. Head Neck.

[CR72] Troch M, Koperek O, Scheuba C, Dieckmann K, Hoffmann M, Niederle B, Raderer M (2010). High efficacy of concomitant treatment of undifferentiated (anaplastic) thyroid cancer with radiation and docetaxel. J Clin Endocrinol Metab.

[CR73] Venkatesh YSS, Ordonez NG, Schultz PN, Hickey RC, Goepfert H, Samaan NA (1990). Anaplastic carcinoma of the thyroid: A clinicopathologic study of 121 cases. Cancer.

[CR74] Wendler J, Kroiss M, Gast K, Kreissl MC, Allelein S, Lichtenauer U, Blaser R, Spitzweg C, Fassnacht M, Schott M, Führer D, Tiedje V (2016). Clinical presentation, treatment and outcome of anaplastic thyroid carcinoma: results of a multicenter study in Germany. Eur J Endocrinol.

[CR75] Yau T, Lo CY, Epstein RJ, Lam AKY, Wan KY, Lang BH (2008). Treatment outcomes in anaplastic thyroid carcinoma: survival improvement in young patients with localized disease treated by combination of surgery and radiotherapy. Ann Surg Oncol.

[CR76] Zheng S, Xu Z, Wei Y, Zeng M, He J (2013). Effect of intraoperative neuromonitoring on recurrent laryngeal nerve palsy rates after thyroid surgery—A meta-analysis. J Formos Med Assoc.

[CR77] Zivaljevic V, Tausanovic K, Paunovic I, Diklic A, Kalezic N, Zoric G, Sabljak V, Vekic B, Zivic R, Marinkovic J, Sipetic S (2014). Age as a prognostic factor in anaplastic thyroid cancer. Int J Endocrinol.

