# Supplementary material for: Role of surgery to the primary tumor in metastatic anaplastic thyroid carcinoma: pooled analysis and SEER-based study
Source: J Cancer Res Clin Oncol. 2022 Aug 12;149(7):3527–47. doi: 10.1007/s00432-022-04223-7 (PMC10314859; doi:10.1007/s00432-022-04223-7)

Supplementary figures


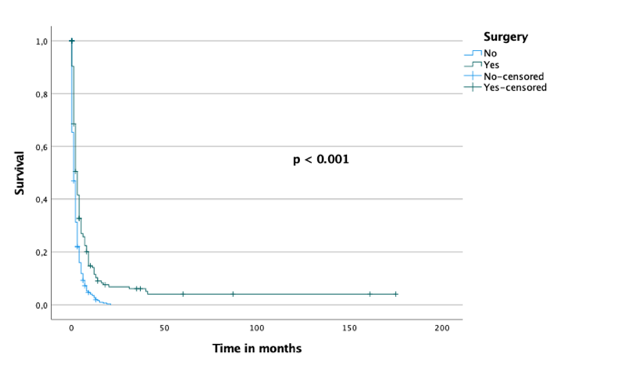

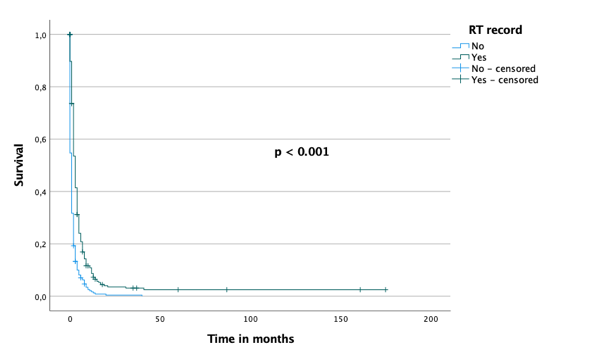

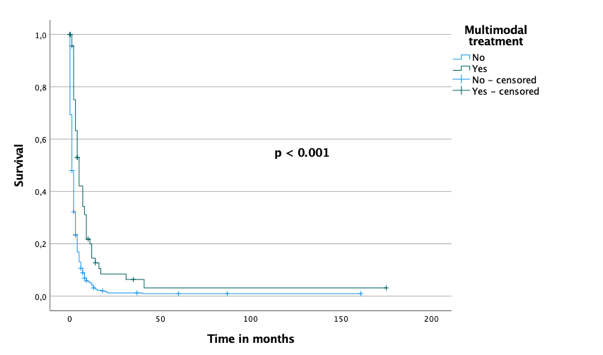
Figures 1A-F. Kaplan-Meier curves of prognostic factors for OS in the whole SEER cohort (n=617)


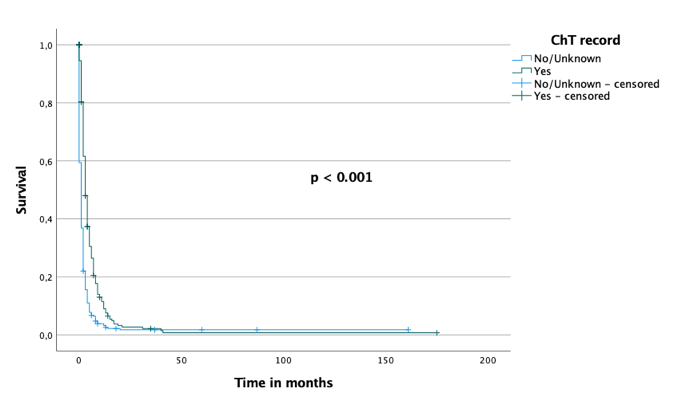


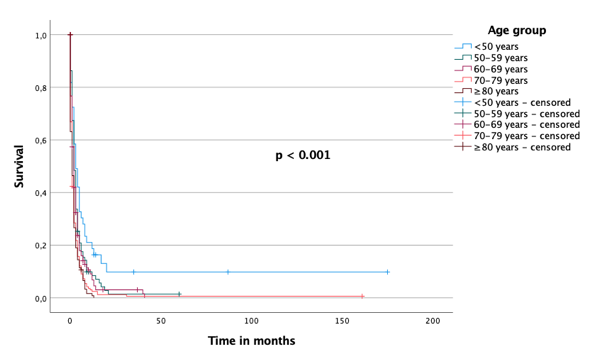

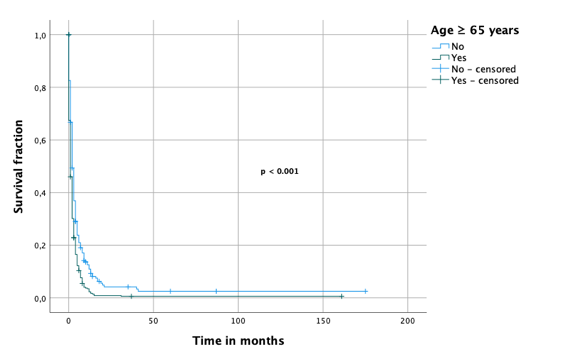


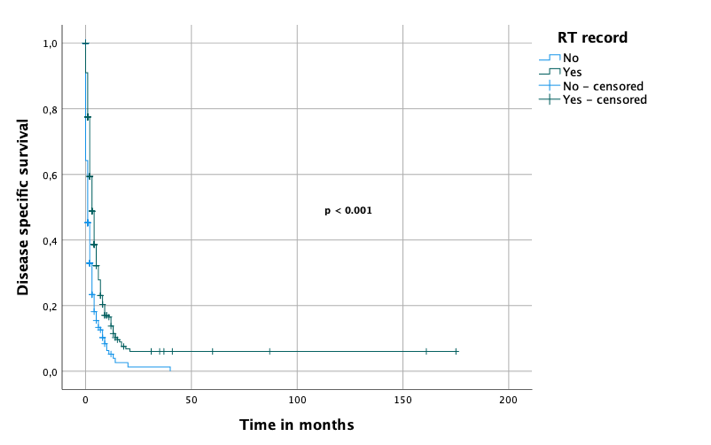

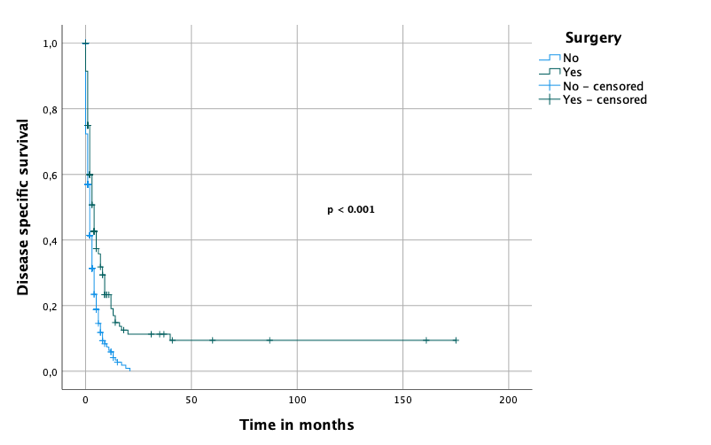
Figures 2A-F. Kaplan-Meier curves of prognostic factors for DSS in the whole SEER cohort (n=617)


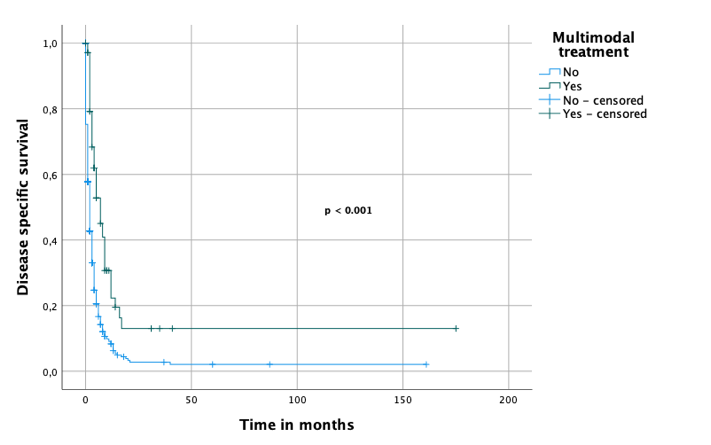

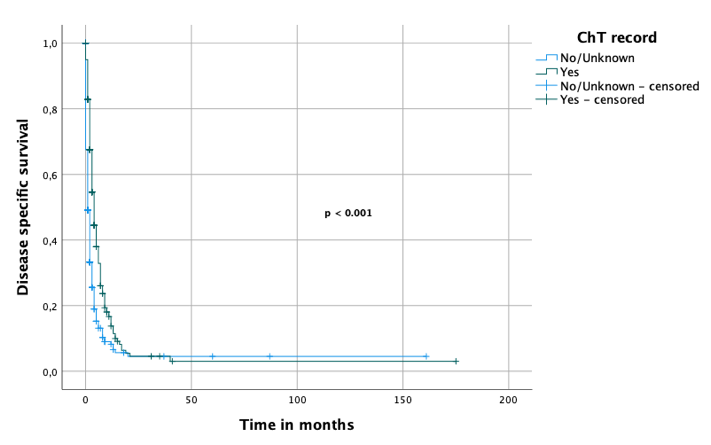


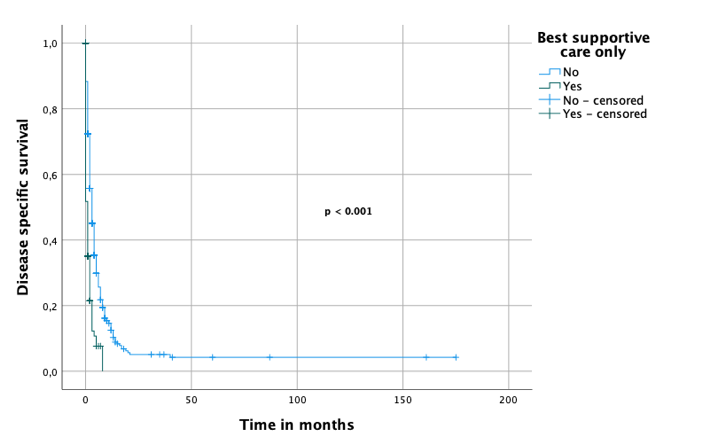

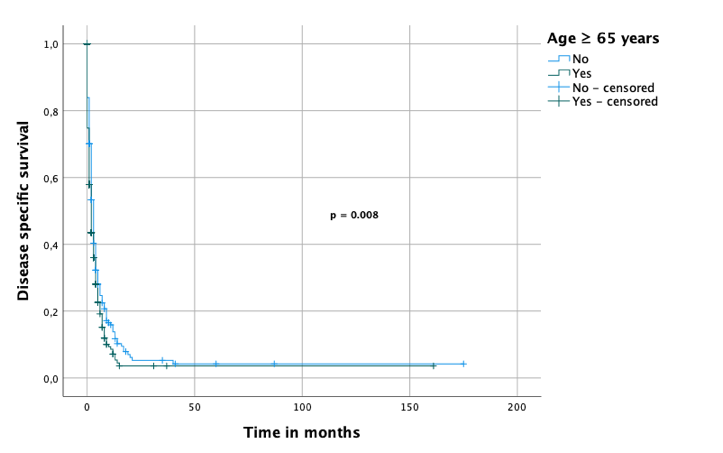


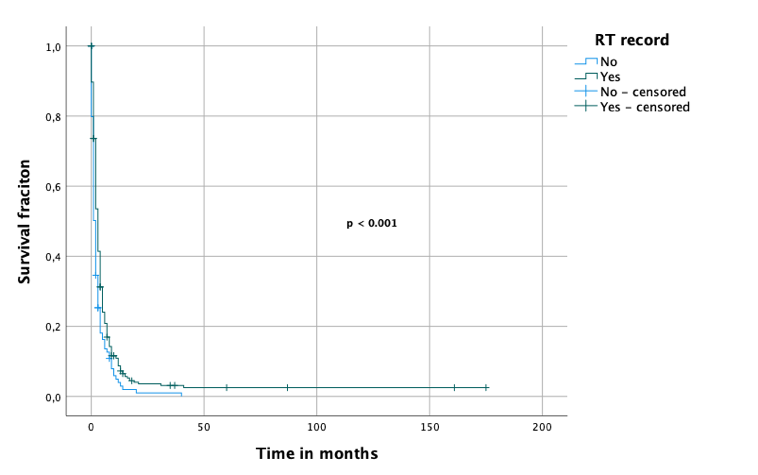

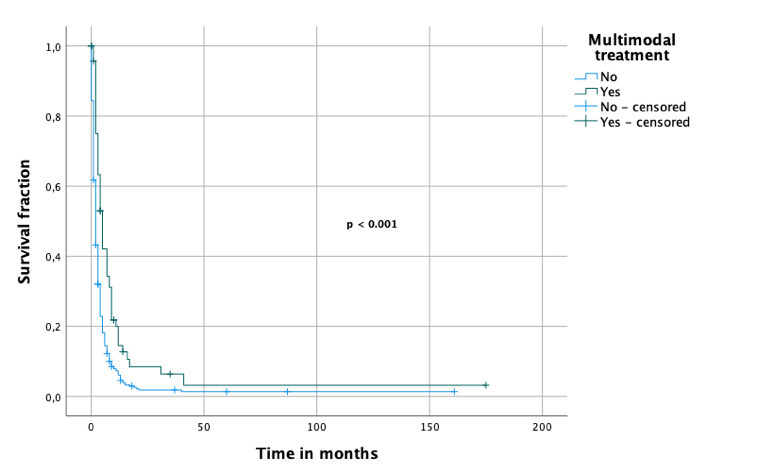

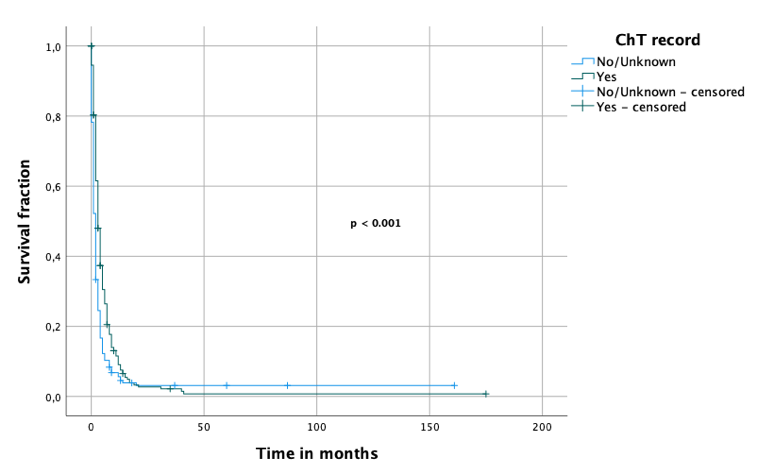

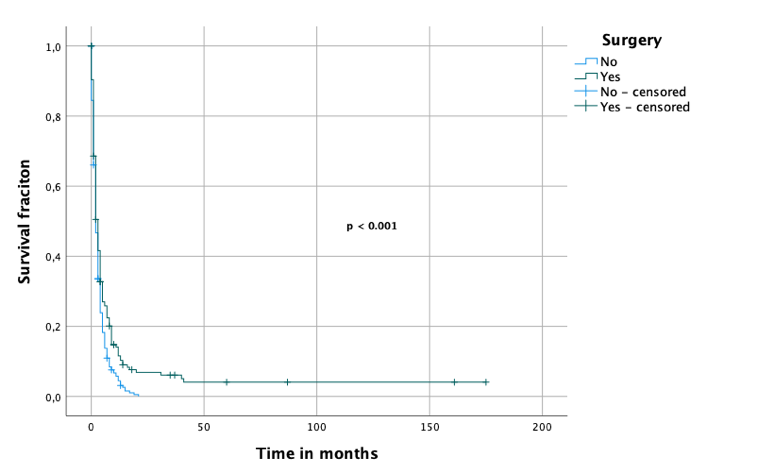
Figures 3A-E. OS of patients from the SEER cohort undergoing any tumor directed treatment


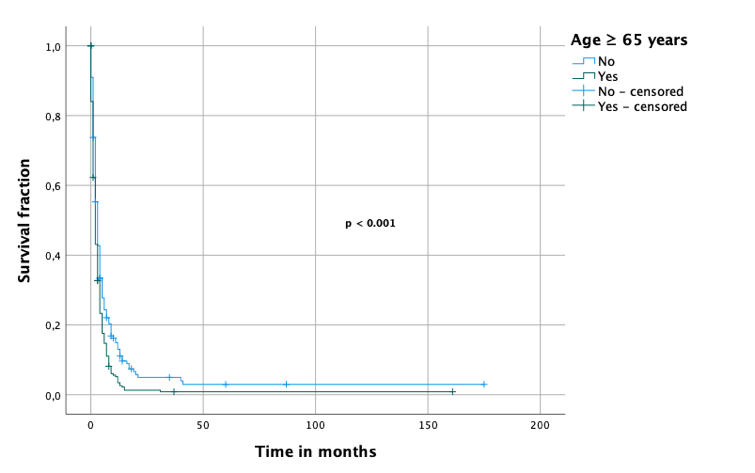


Figure 4A-I. OS of operated cohort from the SEER database


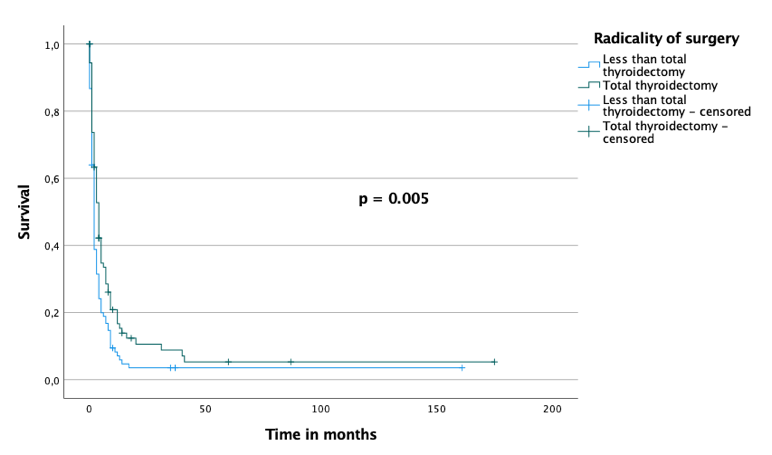

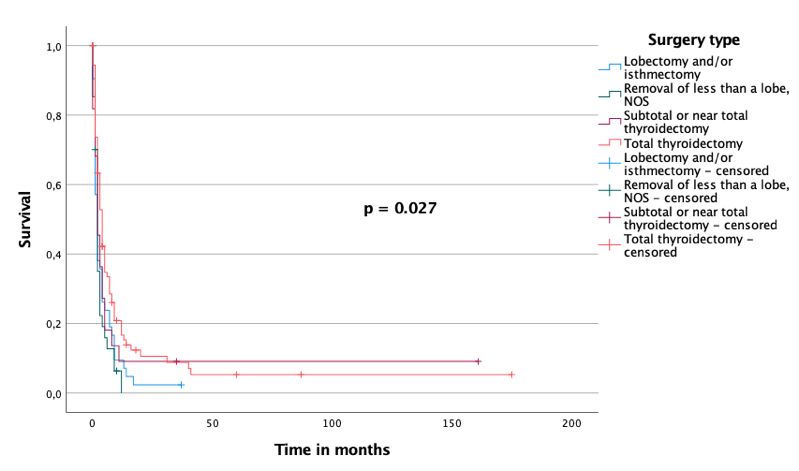


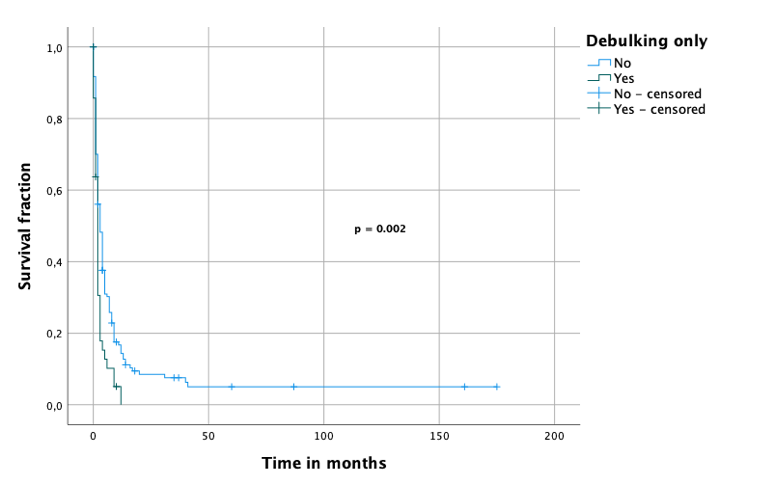

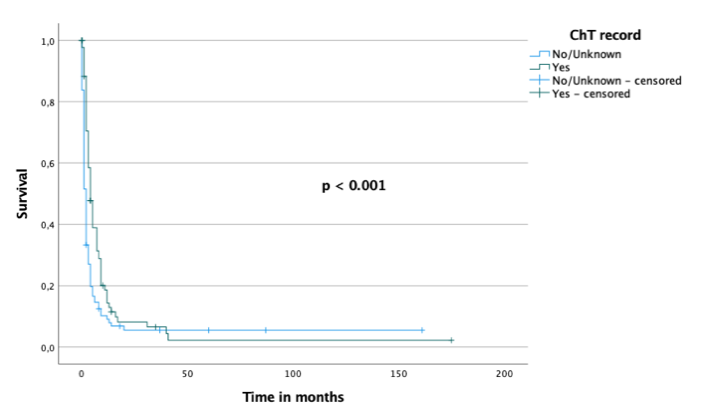

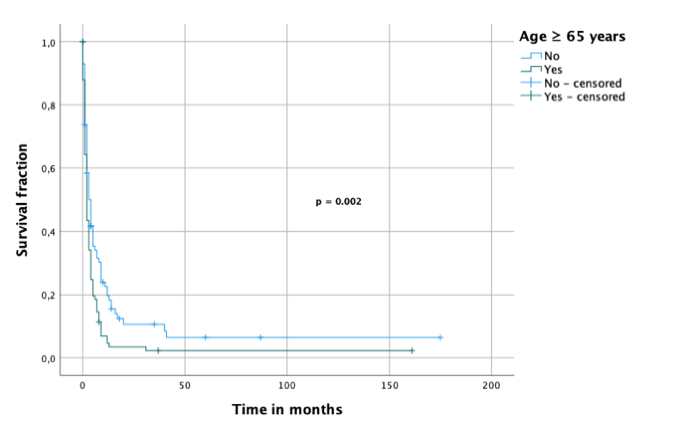

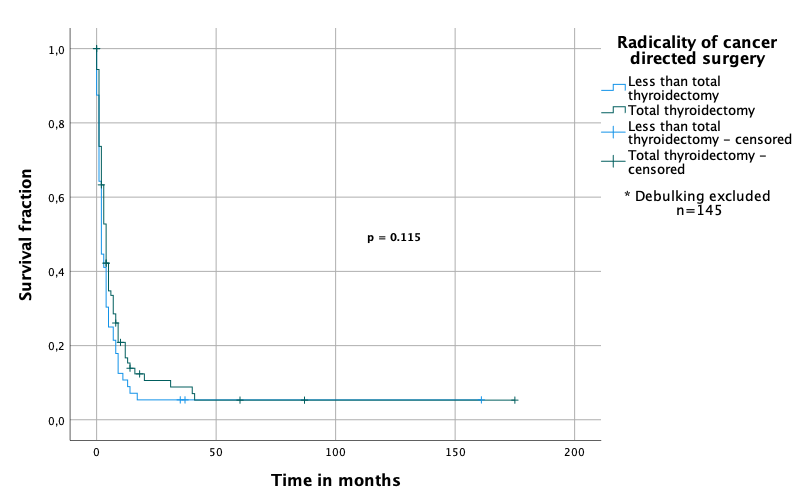

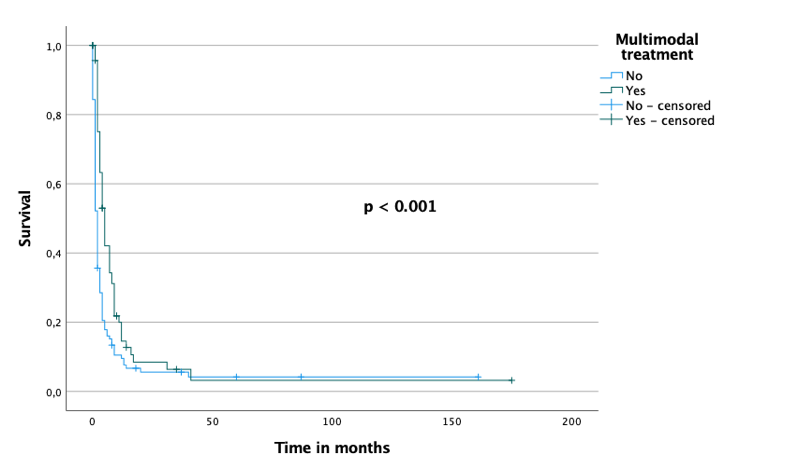

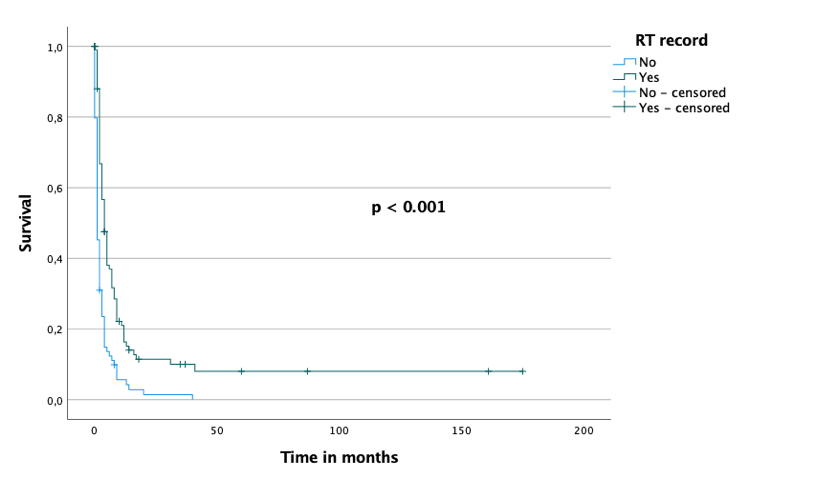

Supplement: Supplementary file 1 — Supplementary file1 (DOCX 31016 KB) [file 432_2022_4223_MOESM1_ESM.docx]
